# Supplementary material for: BioE3 identifies specific substrates of ubiquitin E3 ligases
Source: Nat Commun. 2023 Nov 23;14:7656. doi: 10.1038/s41467-023-43326-8 (PMC10667490; doi:10.1038/s41467-023-43326-8)
Supplement: Supplementary file 1 — Supplementary Information [file 41467_2023_43326_MOESM1_ESM.pdf]

BioE3 identifies specific substrates of ubiquitin E3 ligases

Barroso-Gomila *et al*

SUPPLEMENTARY INFORMATION

Supplementary Note 1:

Use of the wild type AviTag leads to general and nonspecific biotinylation

To evaluate the spatial-specificity of the labelling obtained using the bioUb strategy, we first fused the bio^{WHE} tag to a version of Ub that is not processable by DUBs (Ubnc for Ub non-cleavable, L73P mutation)[1]. This would avoid any recycling of locally pre-labelled bio^{WHE}Ubncs. Although this is not ideal and will disrupt regulation by DUBs, the bioUbnc is inducibly expressed for 24hrs only and likely does not reach levels of endogenous Ub. We generated HEK293FT and U2OS double stable cell lines for TRIPZ-bio^{WHE}Ubnc (puromycin-resistant) together with BirA alone or CEP120-BirA (blasticidin-resistant). The CEP120-BirA fusion protein localizes well to the centrosome. Doxycycline induction of bio^{WHE}Ubncs and 16 hours of biotin labelling yielded strong biotinylation of bio^{WHE}Ubncs in both conditions, with identical biotinylation patterns (Supplementary Fig. 1a). These results suggest that at longer biotin labelling times, BirA labels bio^{WHE}Ubncs in a general way, independently of their localization. To further evaluate the subcellular localization of the signal, we performed immunostainings on the U2OS double stable cell lines (with both inducible bio^{WHE}Ubnc and constitutive CEP120-BirA). The correct localization of CEP120-BirA was first confirmed, showing localization that correspond to centrosomes (single/paired dots, adjacent to or overlying the nucleus), while BirA alone localized to the nucleus and the cytoplasm (Supplementary Fig. 1b and c). We first observed that even without any biotin addition, CEP120-BirA and BirA carrying cell lines biotinylate bio^{WHE}Ubncs in a strong and general way when cultured with normal Fetal Bovine Serum (FBS) containing media (typically supplemented at 10%, which represents approximately 2 nM of biotin [2]; Supplementary Fig. 1b). However, when removing the biotin by dialyzing the serum, only the typical endogenous carboxylase biotinylation-derived streptavidin signal was observed (recognizable by the residual mitochondrial-like staining, Supplementary Fig. 1b). Strikingly, even when using dialyzed serum, biotin pulses as short as 2 minutes already showed a general labelling in the U2OS TRIPZ-bio^{WHE}Ubnc / BirA double stable cell line, whereas in the case of CEP120-BirA, the signal was localized at the centrosome (Supplementary Fig. 1c). Nevertheless, at longer timings, while CEP120-BirA remained specifically localized at the centrosome, we observed non-specific, general streptavidin localization after only 1 hour of biotin labelling (Supplementary Fig. 1c). Altogether, these results show that using dialyzed FBS enables the control of biotin labelling timings and that biotinylation of the bio^{WHE} tag is unspecific to the localization of BirA enzyme even at short labelling timings, probably due to the high affinity

between BirA and the bio^{WHE} tag. In other words, non-conjugated bio^{WHE}Ub is likely attracted to BirA and gets labelled, and incorporates into any substrates, primarily in the nucleus where abundant ubiquitination occurs. Further improvements were therefore required to achieve the specificity needed for BioE3 strategy. All the following experiments were performed using media supplemented with dialyzed (biotin-depleted) FBS.

Supplementary Note 2:

NEDD4 E3 ligase activation enhances BioE3 activity

We evaluated NEDD4 BioE3 efficiency by performing standard BioE3 experiments comparing BirA-NEDD4^{WT} and its transthiolation deficient BirA-NEDD4^{CA} mutant. We observed very low and comparable levels of BioE3 biotinylation activity when using either BirA-NEDD4^{WT} or BirA-NEDD4^{CA} (Supplementary Fig. 10a, Biotin blot). This lack of BioE3 activity is probably due to the fact that NEDD4 ligases need to be activated, through EGF/FGF or intracellular calcium uptake, to stimulate their E3 ligase activity [7-9]. We thus evaluated NEDD4 Ub BioE3 activity in the U2OS – TRIPZ-bio^{GEF}Ubnc cell line, treating the cells with ionomycin and $CaCl_2$ to induce intracellular calcium uptake and with MG132 to inhibit proteasomal degradation. We observed that, at basal-conditions, inactivated NEDD4 localizes to the cytoplasm as reported previously, showing some BioE3 activity (Supplementary Fig. 10b). However, upon NEDD4 activation through ionomycin treatment, we observed that NEDD4 localized to the plasma membrane as well as to cytoplasmic structures that might correspond to vesicles, with high BioE3 activity that was accumulated upon proteasomal inhibition (Supplementary Fig. 10b). Thus, NEDD4 ligase needs to be activated to perform efficient BioE3 experiments. We therefore decided to mimic this activation process by mutating its autoinhibitory C2 domain. We generated a BirA-NEDD4$^{\Delta C2}$ version that lacks the entire C2 domain, as well as a BirA-NEDD4^{3M} version in which three key amino acids that participate in NEDD4-closed conformation were mutated (I36A, L37A and Y604A) [10]. We evaluated NEDD4 Ub BioE3 using BirA- NEDD4^{WT}, NEDD4^{CA}, NEDD4^{3M} or NEDD4$^{\Delta C2}$ and observed that the hyper-activated versions NEDD4^{3M} and NEDD4$^{\Delta C2}$ showed enhanced BioE3 activity (Supplementary Fig. 10c). These results show that activation of NEDD4 is essential to detect its E3 ligase activity.

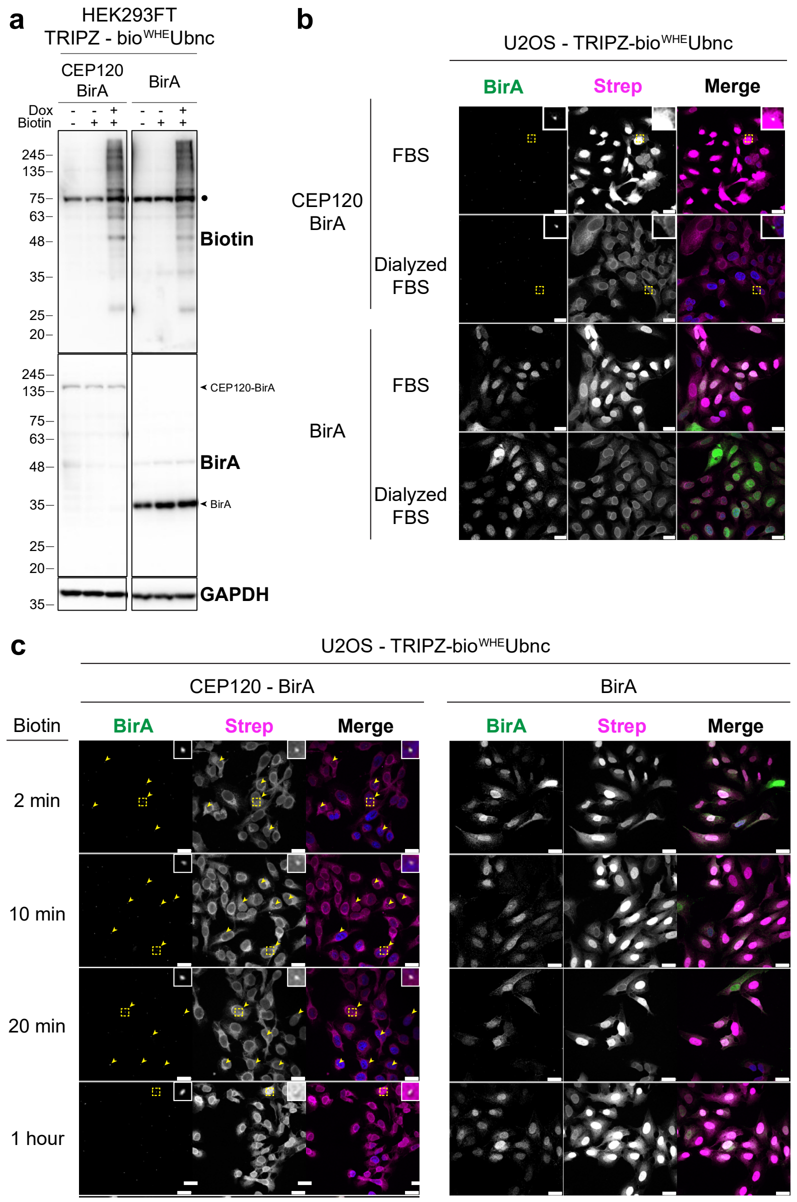

<u>Supplementary Fig. 1:</u> Biotinylation of the wild type AviTag is general and unspecific. (a) Western blot of 293FT stable cell lines expressing TRIPZ-bio^{WHE}Ubnc, together with EFS-BirA or the centrosome-localized CEP120-BirA. Doxycycline (DOX) induction was performed at 1 µg/ml for 24 hours and biotin supplementation at 50 µM for 16 hours. Similar general labelling of bio^{WHE}Ubncs was observed in both conditions. Dot indicates endogenous carboxylases that are biotinylated constitutively by the cell. Results are representative of three independent experiments performed on the same stable cell lines. Molecular weight markers are shown to the left of the blots in kDa. Source data are provided in the Source Data file. **(b-c)** Confocal microscopy of U2OS stable cell lines expressing TRIPZ-bio^{WHE}Ubnc, together with EFS-BirA or the centrosome-localized CEP120-BirA. Cells were pre-incubated in biotin-free dialyzed FBS-containing media or regular FBS-containing media for 24 hours prior to doxycycline induction at 1 µg/ml for 24 hours. No biotin was added in **(b)**. Cells cultured in dialyzed FBS only showed background originated from carboxylase biotinylation-derived streptavidin signal, while cells cultured in normal FBS showed general, unspecific labelling of bio^{WHE}Ubnc. 50 µM of biotin was added to dialyzed FBS-containing media cultured cells at indicated time-points in **(c)**. Colocalization of streptavidin and CEP120-BirA signals was observed at short biotin pulses (yellow arrowheads), while general unspecific labelling was observed at 1 hour of biotin treatment. Yellow dotted-line squares show the selected colocalization events for digital zooming. Nuclei are stained with DAPI (blue), biotinylated material with fluorescent streptavidin (Strep, magenta), and BirA with specific antibody (green). Black and white panels show the green and magenta channels individually. Results are representative of three independent experiments. Scale bar: 25 µm.

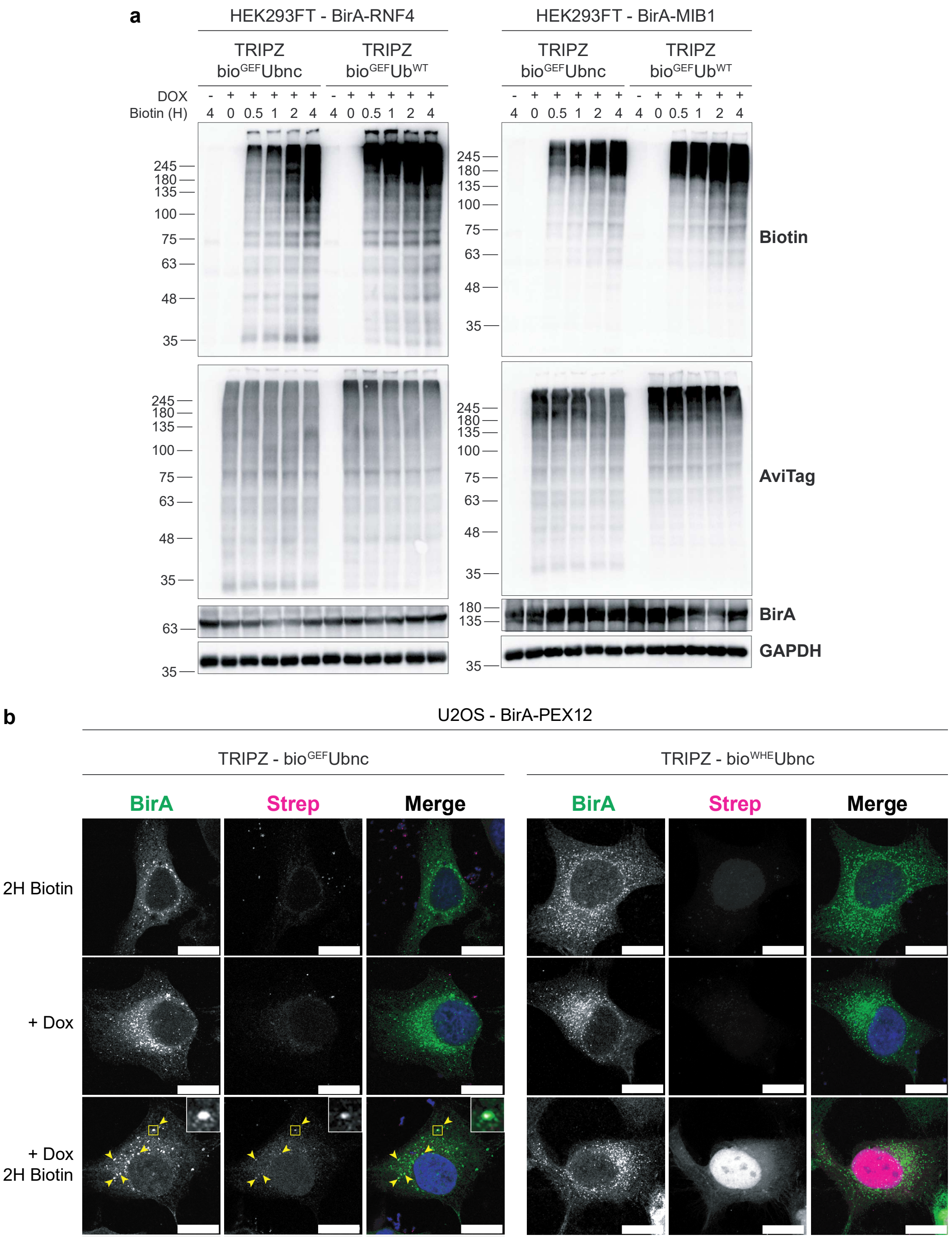

Supplementary Fig. 2: Low affinity bio^{GEF} tag enables BioE3 studies. (a) Western blot of BioE3 experiment performed on HEK293FT stable cell lines expressing TRIPZ-bio^{GEF}Ubnc or TRIPZ-bio^{GEF}Ub^{WT} and transfected with EFS-BirA-RNF4^{WT} or EFS-BirA-MIB1^{WT}. Molecular weight markers are shown to the left of the blots in kDa. Cells were pre-incubated in dialyzed FBS-containing media prior to transfections, doxycycline (DOX) induction at 1 µg/ml for 24 hours and biotin supplementation at 50 µM for indicated time-points. Data are representative of 2 independent transfection experiments with similar results. Source data are provided in the Source Data file. **(b)** Confocal microscopy of U2OS stable cell lines expressing TRIPZ-bio^{WHE}Ubnc or TRIPZ-bio^{GEF}Ubnc transfected with EFS-BirA-PEX12. Cells were pre-incubated in biotin-free dialyzed FBS-containing media for 24 hours prior to transfection and doxycycline induction at 1 µg/ml for 24 hours. 50 µM of biotin was added for 2 hours. Correct colocalization of streptavidin and BirA-PEX12 signals was observed in peroxisomes for bio^{GEF}Ubnc (yellow arrowheads), while general unspecific labelling was detected for bio^{WHE}Ubnc. Dotted yellow line squares show the selected colocalization event for digital zooming. Nuclei are stained with DAPI (blue), biotinylated material with fluorescent streptavidin (Strep, magenta), and BirA with specific antibody (green). Black and white panels show the green and magenta channels individually. Scale bar: 10 µm.

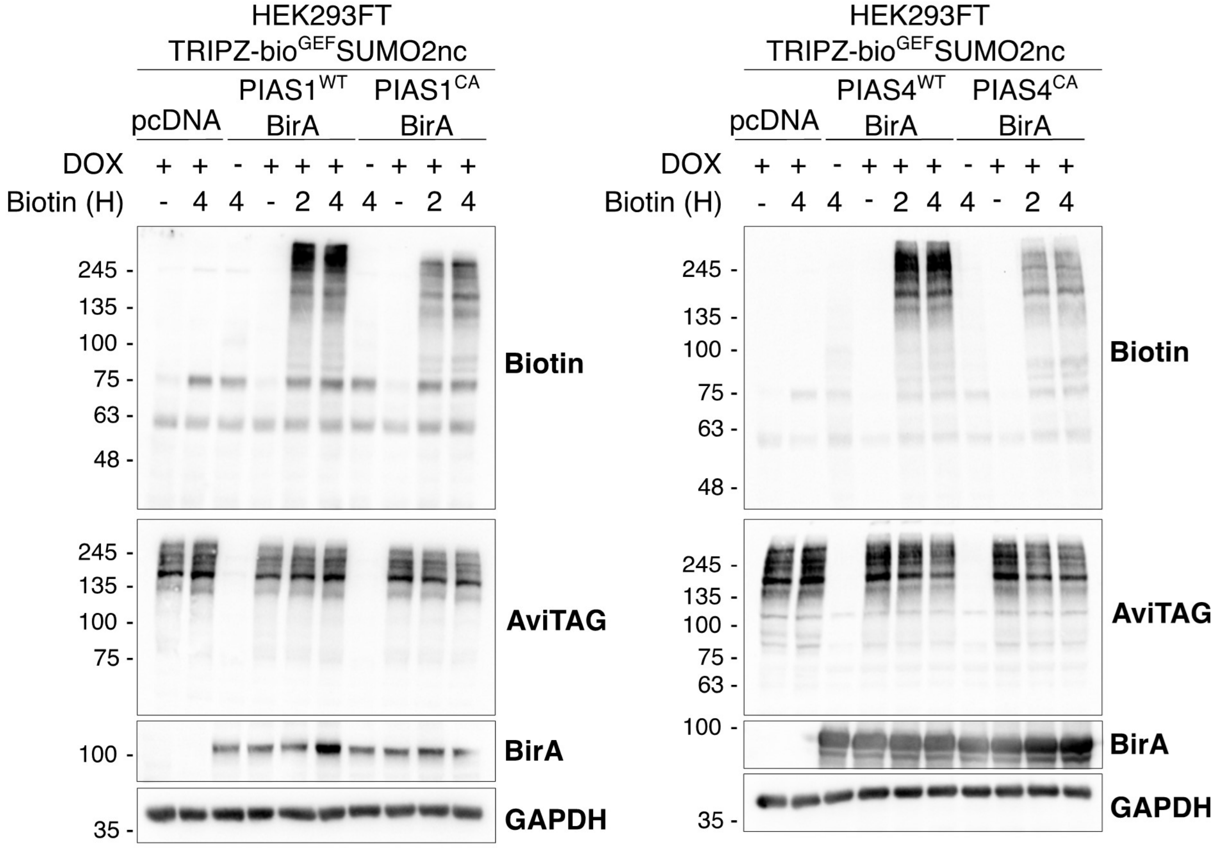

<u>Supplementary Fig. 3:</u> BioE3 labels substrates of SUMO E3 ligases. Western blot of BioE3 experiment performed on HEK293FT stable cell line expressing TRIPZ-bio^{GEF}SUMO2nc and transfected with CMV-PIAS1^{WT}-BirA or CMV-PIAS1^{CA}-BirA (left) and CMV-PIAS4^{WT}-BirA or CMV-PIAS4^{CA}-BirA (right). All BioE3 experiments were performed by pre-incubating the cells in dialyzed FBS-containing media prior to transfections, doxycycline (DOX) induction at 1 µg/ml for 24 hours and biotin supplementation at 50 µM for indicated time points. Molecular weight markers are shown to the left of the blots in kDa, antibodies used are indicated to the right. Data are representative of 2 independent transfection experiments with similar results. These samples were prepared for western analysis and not scaled-up for mass spectrometry analysis. Source data are provided in the Source Data file.

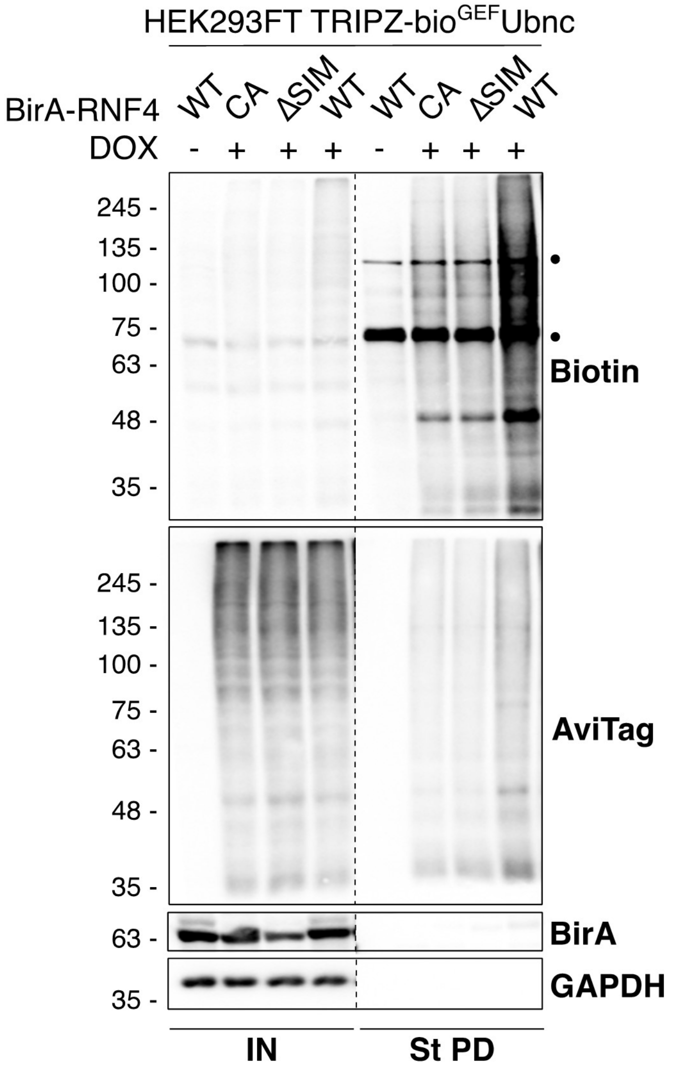

<u>Supplementary Fig. 4:</u> BioE3 labels substrates of RNF4. Western blot of BioE3 experiment performed on HEK293FT stable cell line expressing TRIPZ-bio^{GEF}Ubnc and transfected with EFS-BirA-RNF4^{WT}, BirA-RNF4^{CA} or BirA-RNF4$^{\Delta SIM}$ (related to Fig. 4). IN: input; St PD: streptavidin pull-down. Dotted line indicates a cut in the same blot. Dots indicate endogenously biotinylated carboxylases. Molecular weight markers are shown to the left of the blots in kDa, antibodies used are indicated to the right. Data are representative of 3 independent transfection experiments with similar results. These samples are from scaled-up experiments for mass spectrometry analysis, and representative of the three independent replicates. Source data are provided in the Source Data file.

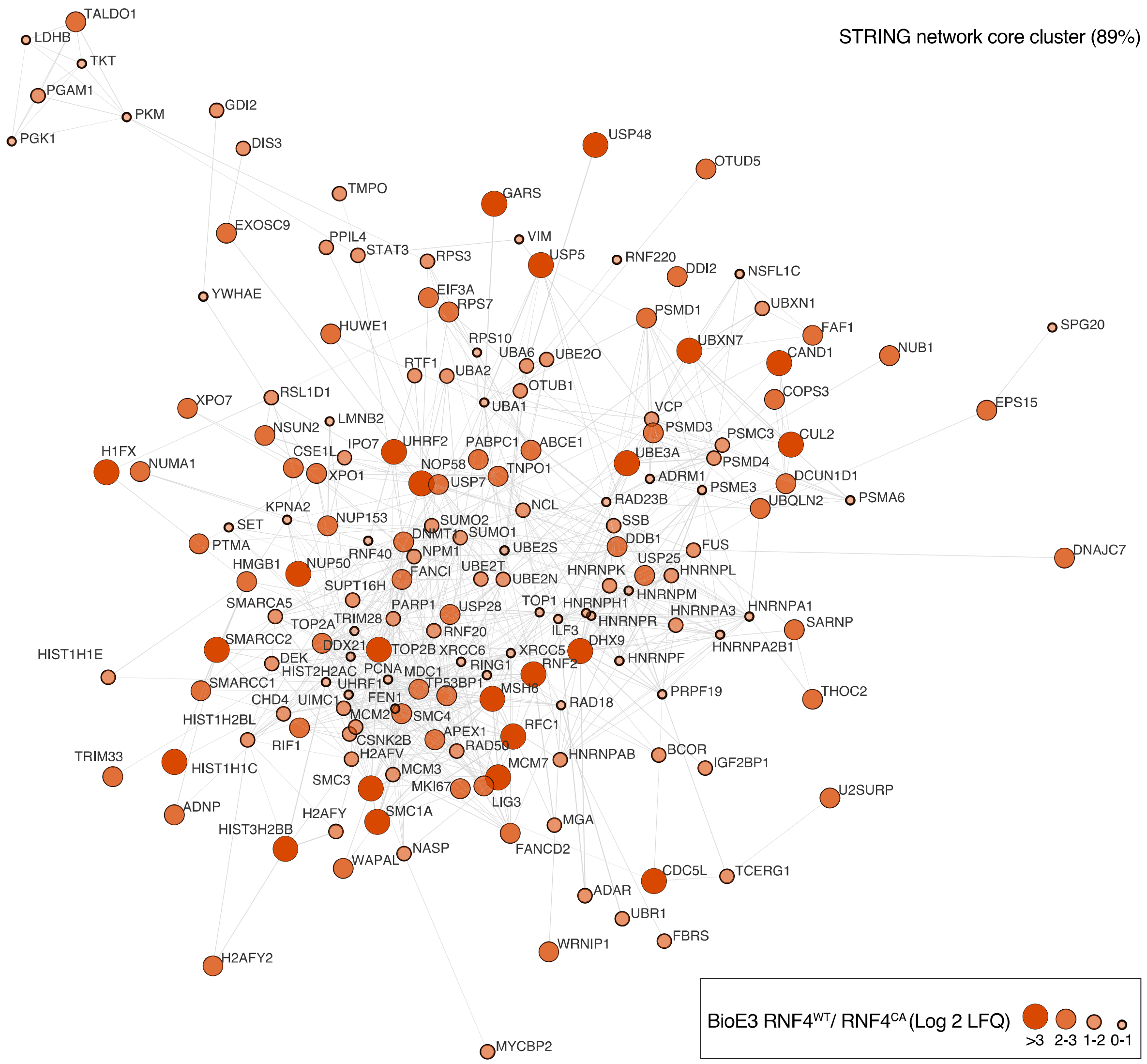

<u>Supplementary Fig. 5:</u> RNF4 Ub substrates form a high interconnected core-cluster. STRING network analysis of bio^{GEF}Ubnc RNF4 targets defined in Fig. 4a, showing a high interconnected network composed of the 89% of the proteins. Color, transparency and size of the nodes were discretely mapped to the Log2 enrichment value as described.

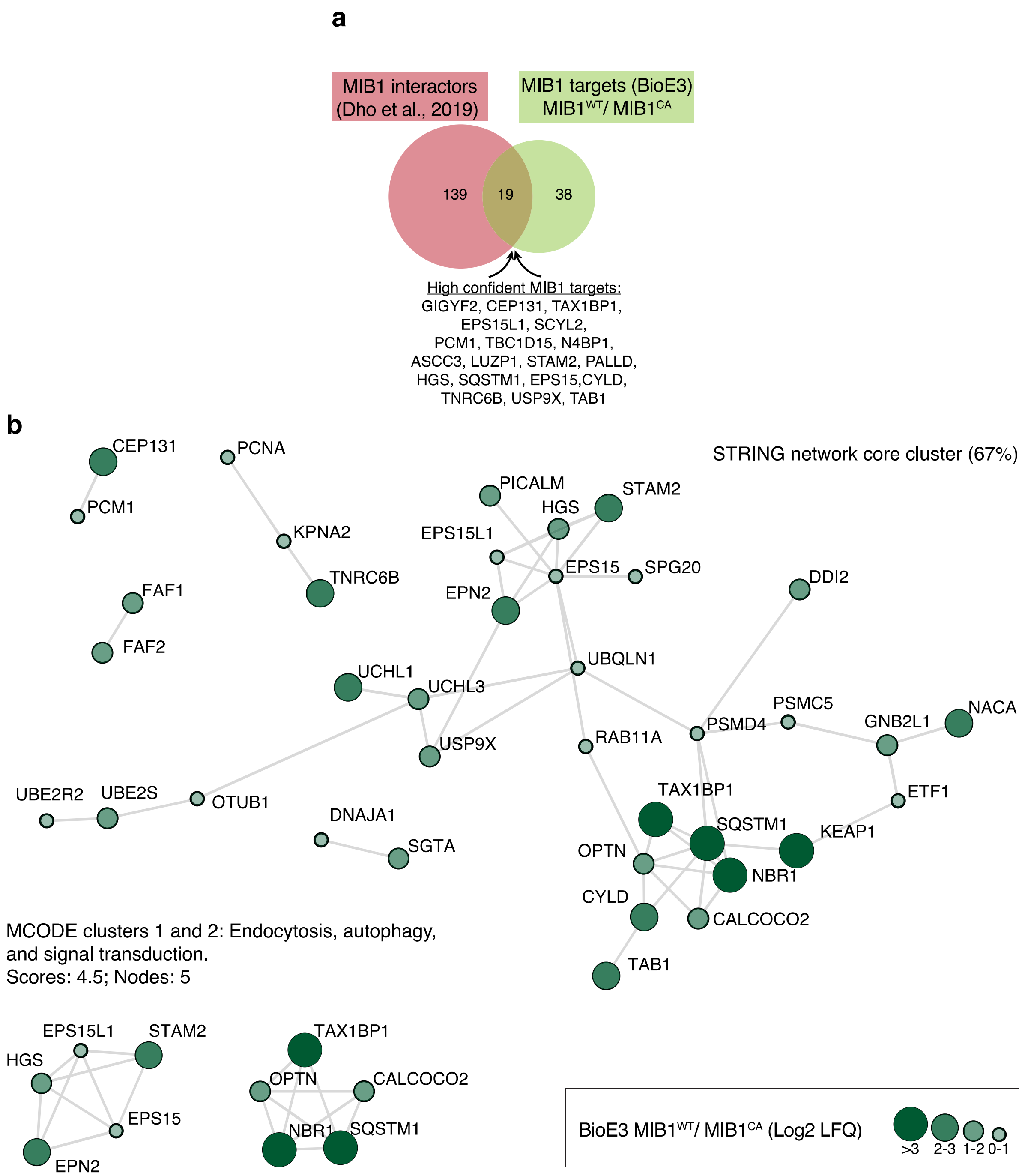

<u>Supplementary Fig. 6:</u> Comparison and validations of MIB1 Ub targets. (a) Venn diagram showing the Ub targets of MIB1 (comparison of the BioE3 MIB1^{WT}/MIB1^{CA} targets in Fig. 6c) and the MIB1 interactome (MIB1 BioID from Dho *et al.* [3]). Comparisons data are provided in Supplementary Data 3. **(b)** STRING network analysis of bio^{GEF}Ubnc MIB1 targets defined in Fig. 6c, showing a high interconnected network composed of the 71% of the proteins. Highly interconnected sub-clusters were derived and characterized using MCODE. Color, transparency and size of the nodes were discretely mapped to the Log2 enrichment value as described.

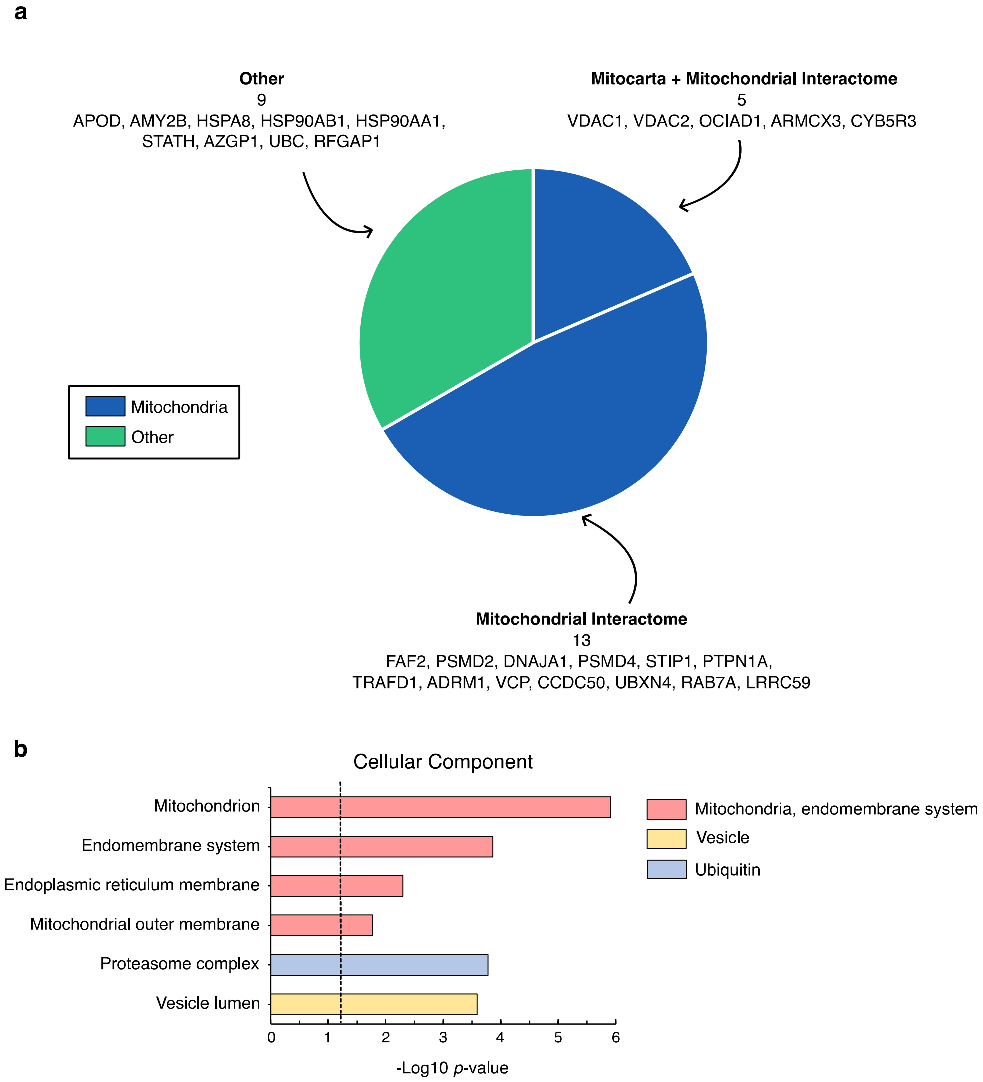

<u>Supplementary Fig. 7:</u> MARCH5 BioE3 identifies mitochondrial proteins. (a) Comparison of the BioE3 MARCH5 targets identified in Fig. 7e with Mitocarta [4], an inventory of mitochondrial proteins, and the mitochondrial proximity interaction network defined by Antonicka *et al.* [5]. Comparisons data are provided in Supplementary Data 5. **(b)** Gene ontology analysis of the MARCH5 targets defined in Fig. 7e. Statistical enrichment analysis was performed using Fisher's one-tailed test with g:SCS correction for multiple comparisons. Depicted cellular components were significantly enriched. Dotted line represents the threshold of the *p*-value (0.05). Data are provided as Supplementary Data 6.

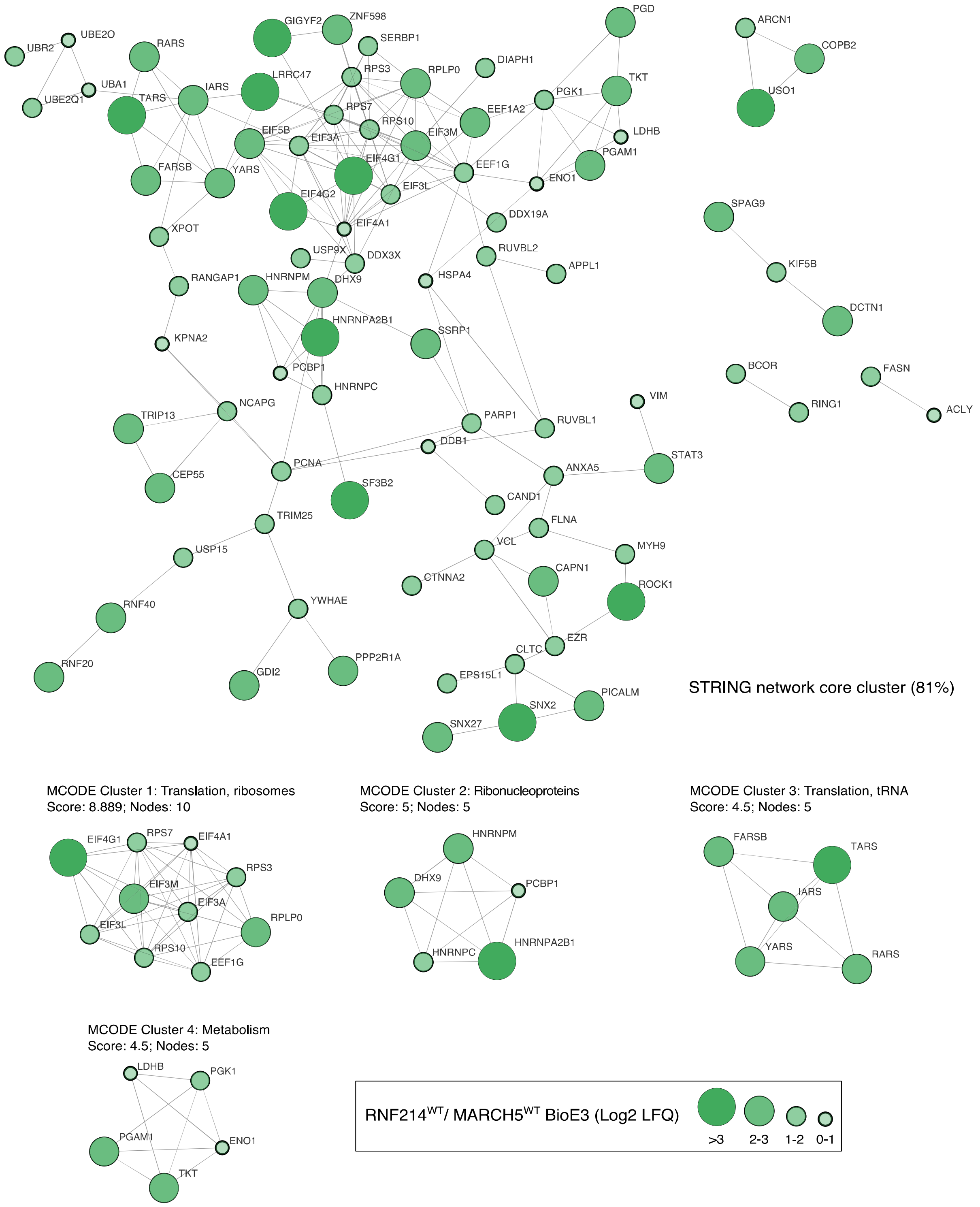

<u>Supplementary Fig. 8:</u> RNF214 targets are involved in translation, intracellular trafficking and cytoskeleton. STRING network analysis of the RNF214 targets defined in Fig. 7e, showing a high interconnected network composed of the 81% of the proteins. Highly interconnected sub-clusters were derived from the core-cluster using MCODE. Color, transparency and size of the nodes were discretely mapped to the Log2 enrichment value as described.

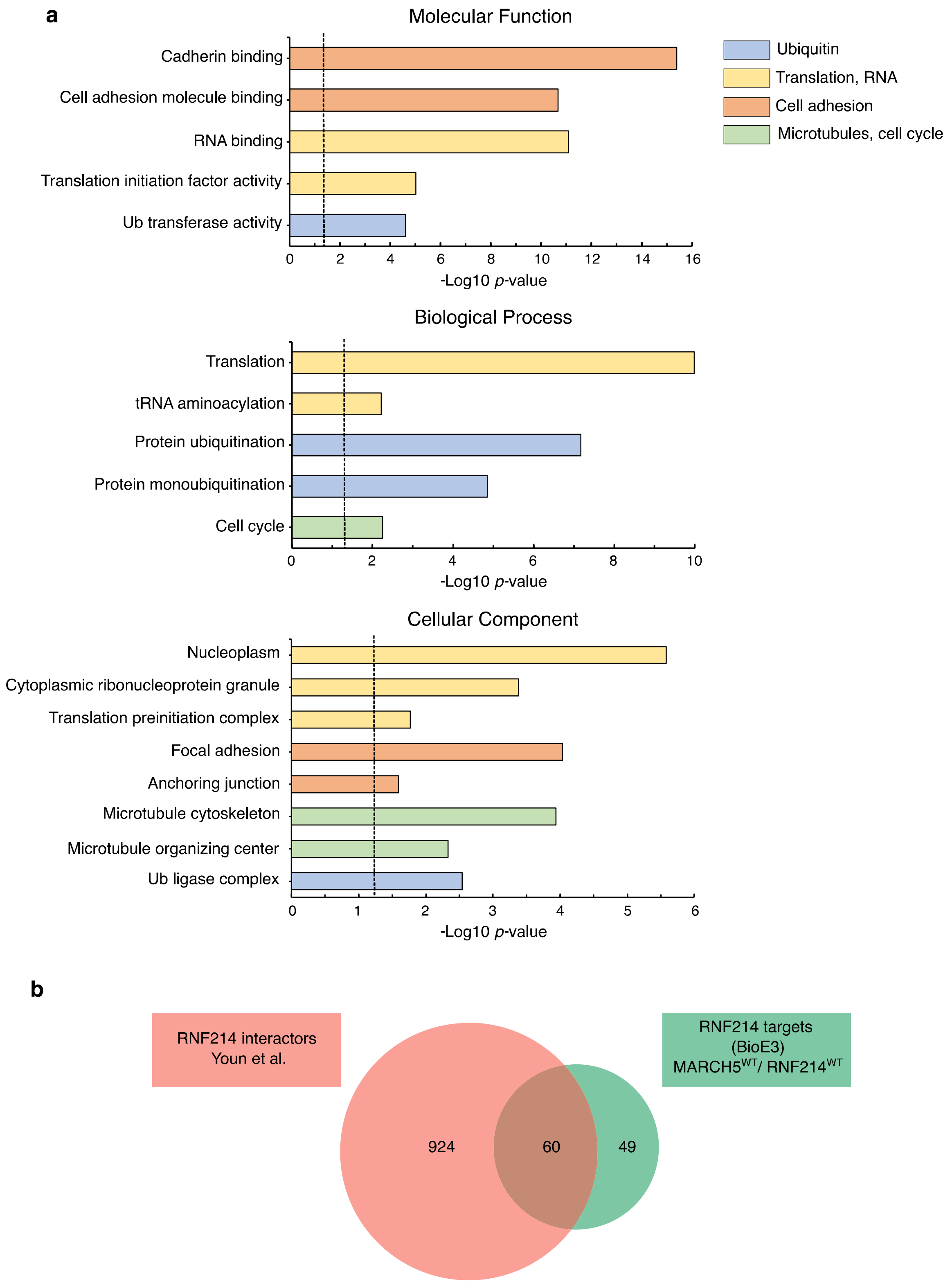

<u>Supplementary Fig. 9:</u> **BioE3 identifies RNF214 targets related to translation, actin cytoskeleton, microtubules and Ub. (a)** Gene ontology analysis of the RNF214 targets defined in Fig. 7e. Statistical enrichment analysis was performed using Fisher's one-tailed test with g:SCS correction for multiple comparisons. Depicted biological processes, molecular functions and cellular components were significantly enriched. Dotted line represents the threshold of the *p*-value (0.05). Data are provided as Supplementary Data 6. **(b)** Venn diagram showing the Ub targets of RNF214 (comparison of the BioE3 $MARCH5^{WT}$/$RNF214^{WT}$ targets in Fig. 7e) and the RNF214 interactome (RNF214 BioID from Youn *et al.* [6]). Comparisons data are provided in Supplementary Data 5.

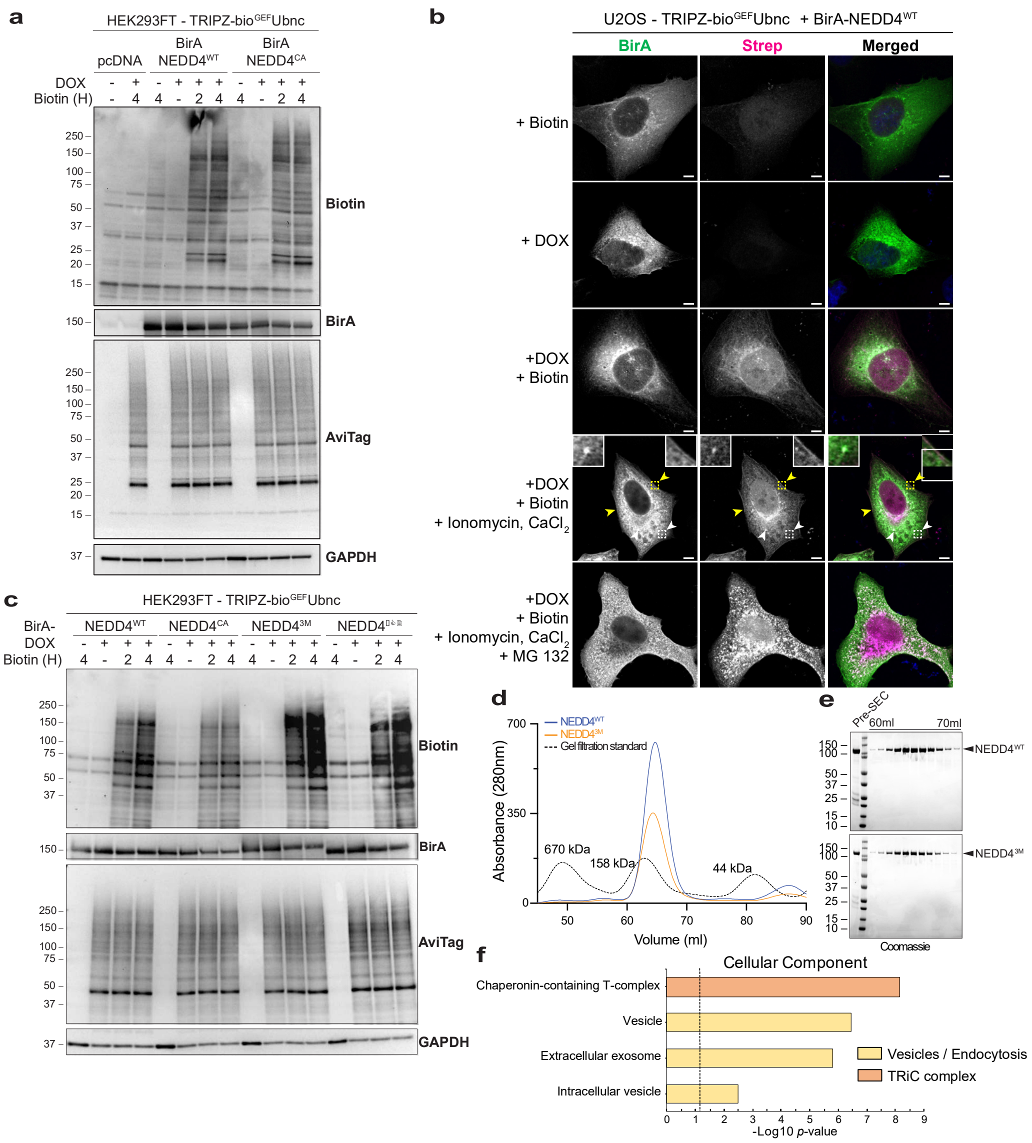

Supplementary Fig. 10: NEDD4 E3 ligase activation enhances BioE3 activity. (a) Western blot of BioE3 experiment performed on 293FT stable cell line expressing TRIPZ-bio^{GEF}Ubnc and transfected with EFS-BirA-NEDD4^{WT} or BirA-NEDD4^{CA}. No efficient BioE3 activity was detected. **(b)** Confocal microscopy of BioE3 experiment performed on U2OS stable cell line expressing TRIPZ-bio^{GEF}Ubnc and transiently transfected with EFS-BirA-NEDD4^{WT}. Cells were also treated with 1 µM ionomycin and 2 mM $CaCl_2$ for 2 hours to induce intracellular calcium uptake and with 10 µM MG132 for 6 hours to inhibit proteasomal degradation. Colocalization of streptavidin and BirA-NEDD4^{WT} was observed at plasma membrane (yellow arrowheads; dotted yellow line squares digital zooming, top right box) and intracellular vesicles (white arrowheads; dotted white line squares digital zooming, top left box) upon calcium-mediated activation of NEDD4^{WT}, and the signal accumulates upon MG132 treatment. Nuclei are stained with DAPI (blue), biotinylated material with fluorescent streptavidin (Strep, magenta), and BirA with specific antibody (green). Black and white panels show the green and magenta channels individually. Scale bar: 8 µm. **(c)** Western blot of BioE3 experiments performed on 293FT stable cell line expressing TRIPZ-bio^{GEF}Ubnc and transiently transfected with EFS-BirA-NEDD4^{WT}, BirA-NEDD4^{CA}, BirA-NEDD4^{3M} or BirA-NEDD4$^{\Delta C2}$. NEDD4 activating mutations showed enhanced BioE3 activity. **(d)** Elution profile on Superdex S200 10/300 of NEDD4^{WT} (blue line), NEDD4^{3M} (yellow line), and gel filtration markers (black dashed line). **(e)** Coomassie-stained SDS-PAGE gel of the fractions related to the NEDD4^{WT} and NEDD4^{3M} peaks, from 60 to 70 ml. **(f)** Gene ontology analysis of the NEDD4 targets defined in Fig. 8g. Statistical enrichment analysis was performed using Fisher's one-tailed test with g:SCS correction for multiple comparisons. Depicted cellular components were significantly enriched. Dotted line represents the threshold of the *p*-value (0.05). Data are provided as Supplementary Data 8. **(a-c)** Results are representative of two independent transfection experiments. Molecular weight markers are shown to the left of the blots in kDa. Source data are provided in the Source Data file.

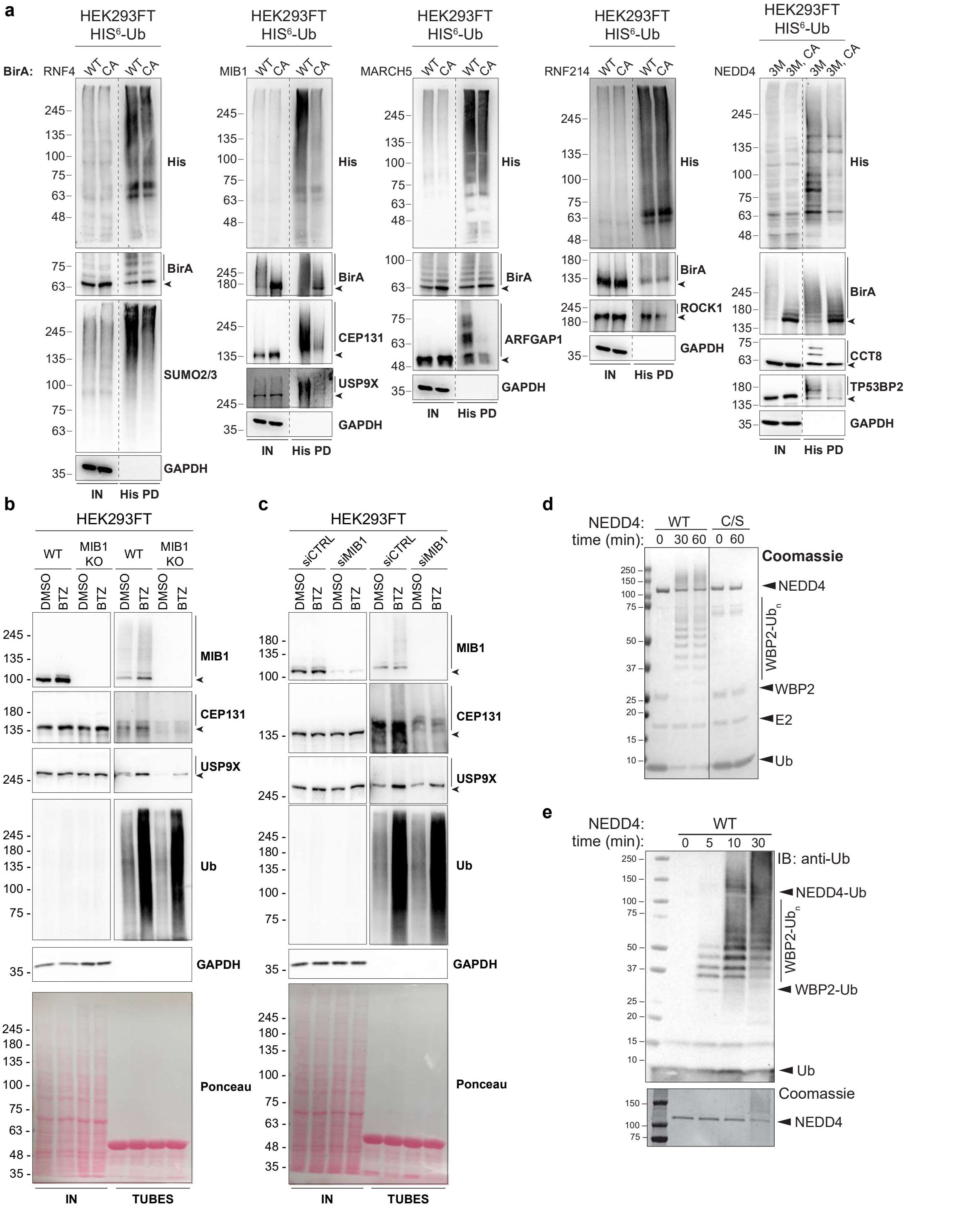

Supplementary Fig. 11: Orthogonal validations of E3 ligase targets identified by BioE3 in this study. (a) His^6-Ub experiments confirming Ub targets of the different E3 ligases used in this study: proteins SUMOylated by SUMO2/3 for RNF4; CEP131 and USP9X for MIB1; ARFGAP1 for MARCH5; ROCK1 for RNF214; CCT8 and TP53BP2 for NEDD4. IN: input; His PD: His^6-Ub pull-down. **(b-c)** CEP131 and USP9X are endogenous Ub targets of MIB1. TUBEs experiments performed on HEK293FT cells comparing **(b)** WT and MIB1KO or **(c)** siCTRL and siMIB1. Cells were also treated with the proteasomal inhibitor bortezomib (BTZ, 400µM, 6 hours). **(d-e)** *In vitro* ubiquitination assay of WBP2 comparing **(d)** NEDD4^{WT} and the catalytically inactive NEDD4^{CS} (Coomassie staining), confirming that WBP2 is a Ub target of NEDD4, and **(e)** western blot of short ubiquitination timings. **(a-e)** Arrowheads and bars point to unmodified and Ub modified proteins, respectively. Molecular weight markers are shown to the left of the blots in kDa. Data are representative of 2 independent experiments with similar results. Source data are provided in the Source Data file.

Supplementary References

1. Bekes, M. *et al.* DUB-resistant ubiquitin to survey ubiquitination switches in mammalian cells. *Cell Rep* **5**, 826-838 (2013).
2. Nesbeth, D. *et al.* Metabolic biotinylation of lentiviral pseudotypes for scalable paramagnetic microparticle-dependent manipulation. *Mol Ther* **13**, 814-822 (2006).
3. Dho, S.E. *et al.* Proximity interactions of the ubiquitin ligase Mind bomb 1 reveal a role in regulation of epithelial polarity complex proteins. *Sci Rep* **9**, 12471 (2019).
4. Rath, S. *et al.* MitoCarta3.0: an updated mitochondrial proteome now with sub-organelle localization and pathway annotations. *Nucleic Acids Res* **49**, D1541-D1547 (2021).
5. Antonicka, H. *et al.* A High-Density Human Mitochondrial Proximity Interaction Network. *Cell Metab* **32**, 479-497 e479 (2020).
6. Youn, J.Y. *et al.* High-Density Proximity Mapping Reveals the Subcellular Organization of mRNA-Associated Granules and Bodies. *Mol Cell* **69**, 517-532 e511 (2018).
7. Lin, Q. *et al.* HECT E3 ubiquitin ligase Nedd4-1 ubiquitinates ACK and regulates epidermal growth factor (EGF)-induced degradation of EGF receptor and ACK. *Mol Cell Biol* **30**, 1541-1554 (2010).
8. Wang, J. *et al.* Calcium activates Nedd4 E3 ubiquitin ligases by releasing the C2 domain-mediated auto-inhibition. *J Biol Chem* **285**, 12279-12288 (2010).
9. Garrone, N.F., Blazer-Yost, B.L., Weiss, R.B., Lalouel, J.M. & Rohrwasser, A. A human polymorphism affects NEDD4L subcellular targeting by leading to two isoforms that contain or lack a C2 domain. *BMC Cell Biol* **10**, 26 (2009).
10. Mari, S. *et al.* Structural and functional framework for the autoinhibition of Nedd4-family ubiquitin ligases. *Structure* **22**, 1639-1649 (2014).
11. Pirone, L. *et al.* A comprehensive platform for the analysis of ubiquitin-like protein modifications using in vivo biotinylation. *Sci Rep* **7**, 40756 (2017).
