# Supplementary material for: BioE3 identifies specific substrates of ubiquitin E3 ligases
Source: Nat Commun. 2023 Nov 23;14:7656. doi: 10.1038/s41467-023-43326-8 (PMC10667490; doi:10.1038/s41467-023-43326-8)
Supplement: Supplementary file 3 — Description of Additional Supplementary Files [file 41467_2023_43326_MOESM3_ESM.pdf]

Description of Additional Supplementary Files

Supplementary Data 1: RNF4 BioE3 LC-MS processed data (sheet 1). Comparison between RNF4 targets identified by BioE3 and PML interactome (sheet 2) and the SUMOylome (sheet 3).

Supplementary Data 2: Gene Ontology analysis of RNF4 targets (sheet 1) and selected GO terms to build the graphics (sheet 2).

Supplementary Data 3: MIB1 BioE3 LC-MS processed data (sheet 1). Comparison between MIB1 targets identified by BioE3 and MIB1 interactome (sheet 2).

Supplementary Data 4: Gene Ontology analysis of MIB1 targets (sheet 1) and selected GO terms to build the graphics (sheet 2).

Supplementary Data 5: MARCH5 *vs* RNF214 BioE3 LC-MS processed data (sheet 1). Comparison between MARCH5 targets identified by BioE3, the mitochondrial interactome and mitocarta (sheet 2). Comparison between RNF214 targets identified by BioE3 and RNF214 interactome (sheet 3).

Supplementary Data 6: Gene Ontology analysis of MARCH5 targets (sheet 1) and selected GO terms to build the graphics (sheet 2). Gene Ontology analysis of RNF214 targets (sheet 3) and selected GO terms to build the graphics (sheet 4).

Supplementary Data 7: $NEDD4^{3M}$ BioE3 LC-MS processed data.

Supplementary Data 8: Gene Ontology analysis of NEDD4 targets (sheet 1) and selected GO terms to build the graphic (sheet 2).

Supplementary Data 9: List of the constructs used in this study. Related to Cloning section of *Methods*. The plasmid name, the resistance for bacterial transformation and amplification (Res), the plasmid backbone in which the DNA of interest (Plasmid insert) was inserted, and notes that explain the backbone as well as the source of the inserted DNA are depicted.

Supplementary Data 10: Oligonucleotides sequences and uses. Related to Cloning section of *Methods*.
