# Supplementary material for: BioE3 identifies specific substrates of ubiquitin E3 ligases
Source: Nat Commun. 2023 Nov 23;14:7656. doi: 10.1038/s41467-023-43326-8 (PMC10667490; doi:10.1038/s41467-023-43326-8)
Supplement: Supplementary file 14 — Reporting Summary [file 41467_2023_43326_MOESM14_ESM.pdf]

Corresponding author(s): Rosa Barrio
James D. Sutherland

Last updated by author(s): Oct 24, 2023

# Reporting Summary

Nature Portfolio wishes to improve the reproducibility of the work that we publish. This form provides structure for consistency and transparency in reporting. For further information on Nature Portfolio policies, see our Editorial Policies and the Editorial Policy Checklist.

## Statistics

For all statistical analyses, confirm that the following items are present in the figure legend, table legend, main text, or Methods section.

| n/a | Confirmed | |
|---|---|---|
| ☐ | ☒ | The exact sample size (*n*) for each experimental group/condition, given as a discrete number and unit of measurement |
| ☐ | ☒ | A statement on whether measurements were taken from distinct samples or whether the same sample was measured repeatedly |
| ☐ | ☒ | The statistical test(s) used AND whether they are one- or two-sided<br>*Only common tests should be described solely by name; describe more complex techniques in the Methods section.* |
| ☒ | ☐ | A description of all covariates tested |
| ☐ | ☒ | A description of any assumptions or corrections, such as tests of normality and adjustment for multiple comparisons |
| ☐ | ☒ | A full description of the statistical parameters including central tendency (e.g. means) or other basic estimates (e.g. regression coefficient) AND variation (e.g. standard deviation) or associated estimates of uncertainty (e.g. confidence intervals) |
| ☐ | ☒ | For null hypothesis testing, the test statistic (e.g. *F*, *t*, *r*) with confidence intervals, effect sizes, degrees of freedom and *P* value noted<br>*Give P values as exact values whenever suitable.* |
| ☒ | ☐ | For Bayesian analysis, information on the choice of priors and Markov chain Monte Carlo settings |
| ☒ | ☐ | For hierarchical and complex designs, identification of the appropriate level for tests and full reporting of outcomes |
| ☒ | ☐ | Estimates of effect sizes (e.g. Cohen's *d*, Pearson's *r*), indicating how they were calculated |

*Our web collection on statistics for biologists contains articles on many of the points above.*

## Software and code

Policy information about availability of computer code

| | |
|---|---|
| Data collection | timsTOF Pro with PASEF, Bruker Daltonics<br>Leica SP8 Lightning confocal mycroscope, 63x Plan ApoChromat NA1.4<br>iBright imaging system, Thermo Fisher |
| Data analysis | MaxQuant v2.2.0.0, Andromeda (RRID: SCR_014485)<br>Perseus v1.6.15.0 (RRID: SCR_015753)<br>ImageJ v2.0.0-rc-69/1.52n (RRID: SCR_003070)<br>GraphPad Prism 9 v.9.3.1 (RRID: SCR_015807)<br>g:Profiler web server version e108_eg55_p17_0254fbf [https://biit.cs.ut.ee/gprofiler/gost]<br>Cytoscape v3.9.1 (RRID: SCR_003032)<br>STRING v1.4.2 (RRID: SCR_005223)<br>MCODE v1.5.1 (RRID: SCR_015828) |

For manuscripts utilizing custom algorithms or software that are central to the research but not yet described in published literature, software must be made available to editors and reviewers. We strongly encourage code deposition in a community repository (e.g. GitHub). See the Nature Portfolio guidelines for submitting code & software for further information.

## Data

Policy information about availability of data

All manuscripts must include a data availability statement. This statement should provide the following information, where applicable:

- Accession codes, unique identifiers, or web links for publicly available datasets
- A description of any restrictions on data availability
- For clinical datasets or third party data, please ensure that the statement adheres to our policy

All data supporting the findings are provided within the paper, the Supplementary Data, the Supplementary Information and the Source Data file. The fasta file of the human proteome (Uniprot filtered reviewed H. sapiens proteome), UP000005640 [https://www.uniprot.org/uniprot/?query=proteome:UP000005640%20reviewed:yes]) was downloaded from Uniprot. In addition, the mass spectrometry proteomics raw data have been deposited to the ProteomeXchange Consortium via the PRIDE partner repository92 with the dataset identifier PXD041685 [https://proteomecentral.proteomexchange.org/cgi/GetDataset?ID=PXD041685]. Processed LC-MS/MS data as well as their corresponding gene ontology source data are provided as Supplementary Data files. The different datasets used in this study are available in:, used for comparisons in this study are available respectively in SUMOylated protein dataset,  DOI: 10.1038/nrm.2016.81 [https://www.nature.com/articles/nrm.2016.81]; MIB1 interactome dataset, DOI: 10.1038/s41598-019-48902-x [https://www.nature.com/articles/s41598-019-48902-x]; Mitocarta dataset, DOI: 10.1093/nar/gkaa1011 [https://www.broadinstitute.org/mitocarta/mitocarta30-inventory-mammalian-mitochondrial-proteins-and-pathways]; mitochondrial interactome dataset, DOI: 10.1016/j.cmet.2020.07.017 [https://www.cell.com/cell-metabolism/fulltext/S1550-4131(20)30412-5?_returnURL=https%3A%2F%2Flinkinghub.elsevier.com%2Fretrieve%2Fpii%2FS1550413120304125%3Fshowall%3Dtrue]; and the RNF214 interactome dataset, DOI: 10.1016/j.molcel.2017.12.020 [https://www.cell.com/molecular-cell/fulltext/S1097-2765(17)30977-2?_returnURL=https%3A%2F%2Flinkinghub.elsevier.com%2Fretrieve%2Fpii%2FS1097276517309772%3Fshowall%3Dtrue]. Source data are provided with this paper.

## Human research participants

Policy information about studies involving human research participants and Sex and Gender in Research.

| | |
|---|---|
| Reporting on sex and gender | N/A |
| Population characteristics | N/A |
| Recruitment | N/A |
| Ethics oversight | N/A |

Note that full information on the approval of the study protocol must also be provided in the manuscript.

# Field-specific reporting

Please select the one below that is the best fit for your research. If you are not sure, read the appropriate sections before making your selection.

☒ Life sciences ☐ Behavioural & social sciences ☐ Ecological, evolutionary & environmental sciences

For a reference copy of the document with all sections, see nature.com/documents/nr-reporting-summary-flat.pdf

# Life sciences study design

All studies must disclose on these points even when the disclosure is negative.

| | |
|---|---|
| Sample size | Sample size for LC-MS/MS and corresponding Western Blots was set up to 80.000.000 cells (4*15cm dishes) per replicate. This was determined by measuring the elution efficiency of pull-down experiments (comparing different sample sizes) and by identifying IDs with pilot LC-MS/MS experiments. The sample size was sufficient and efficient in terms of amount of beads/elution volume ratio, as elution volume restricts the sample size that can be loaded into the gel and analyzed by LC-MS/MS. Experiments were performed in triplicates (n=3). For cell imaging, at least 4 independent acquisitions per coverslip were taken and this was sufficient to have the overall picture for each condition. Sample size was determine to 150.000 cells per cover-slip, as his was sufficient to o have the overall picture for each condition, and in terms of transfection efficiency. For other experiments, sample size was determined rationally, determined on the amount of material nedeed for each experiment: pilot protein experiments were performed plating 300 000 cells on 6 well plates per condition, and repeated at least 2 or 3 times. This was sufficient to perform pilot experiments and to have conclusive results. Sample size for Streptavidin pull-down experiments other than LC-MS was setted up to 20.000.000 cells (1*15cm dish) and this was sufficient and efficient in terms of amount of beads/elution volume ratio. For quantification experiments, experiments were performed in triplicates (n=3) to apply statistical tests. Other information on sample sizes for each experiment is described within the figure legends and Methods section. |
| Data exclusions | For LC-MS/MS data analysis, the exclusion criteria were pre-established and are commonly used for improving the confidence of identifications: proteins detected with at least 2 peptides and in at least 2 of the 3 replicates were included. Contaminants, only identified by site and reverse identified proteins were also excluded. |
| Replication | For all LC-MS/MS experiments, three independent replicates per condition were analyzed, and all attempts at replication were successful. For |

| | |
|---|---|
| Replication | the rest, the number of times that each experiment was performed is described in the associated figure legends. All attempts at replication were successful. |
| Randomization | Randomization is not applicable as no patient data is included in the manuscript. |
| Blinding | LC-MS/MS samples were encoded and blindly acquired and analyzed in the facility. Investigators were blinded to group allocation during data collection. For the rest of the experiments, the investigators were not blinded to group allocations. Although samples were always encoded, blinding was not possible for terms of logistics: logical and visual order of samples in a gel for Western Blotting, order of replicates in microscope slices, etc... Blinding in those experiment was not relevant and proper laboratory practices were employed: samples of the same experiment were collected in the same conditions, replicates were performed and proper positive and negative controls were always included. |

# Reporting for specific materials, systems and methods

We require information from authors about some types of materials, experimental systems and methods used in many studies. Here, indicate whether each material, system or method listed is relevant to your study. If you are not sure if a list item applies to your research, read the appropriate section before selecting a response.

## Materials & experimental systems

| n/a | Involved in the study |
|---|---|
| ☐ | ☒ Antibodies |
| ☐ | ☒ Eukaryotic cell lines |
| ☒ | ☐ Palaeontology and archaeology |
| ☒ | ☐ Animals and other organisms |
| ☒ | ☐ Clinical data |
| ☒ | ☐ Dual use research of concern |

## Methods

| n/a | Involved in the study |
|---|---|
| ☒ | ☐ ChIP-seq |
| ☒ | ☐ Flow cytometry |
| ☒ | ☐ MRI-based neuroimaging |

## Antibodies

| | |
|---|---|
| Antibodies used | Goat polyclonal anti-biotin HRP-linked (Cell Signaling; Cat#7075S; RRID: AB_10696897), Rabbit polyclonal anti-BirA (SinoBiological; Cat#11582-T16), Mouse monoclonal anti-BirA (Novus Biologicals; Cat#NBP2-59939), Rabbit polyclonal anti-AviTag (1/1000; Cat#A00674; RRID: AB_915553), Mouse monoclonal anti-His tag (1/1000; Cat#A00186S; RRID: AB_914704), Rabbit polyclonal anti-PCM1 (Cell Signaling; Cat#5213S; RRID: AB_10556960), Mouse monoclonal anti-GAPDH clone 1E6D9 (Proteintech; Cat#60004-1-Ig; RRID: AB_2107436), Rabbit monoclonal anti-alpha-Actinin clone D6F6 (Cell Signaling Cat#6487S; RRID: AB_11179206), Rabbit polyclonal anti-USP9X (Proteintech; Cat#55054-1-AP; RRID: AB_10792932), Rabbit polyclonal anti-CEP131 (Proteintech; Cat#25735-1-AP; RRID: AB_2880216), Mouse monoclonal anti-SUMO2/3 (Proteintech; Cat#67154-1-Ig; RRID: AB_2882451), Rabbit polyclonal anti-PML (Proteintech; Cat#21041-1-AP; RRID: AB_2878799), Rabbit polyclonal anti-ROCK1 (Proteintech; Cat#21850-1-AP; RRID: AB_10953526), Rabbit polyclonal anti-GIGYF2 (Proteintech; Cat#24790-1-AP; RRID: AB_2879727), Rabbit polyclonal anti-CLINT1 (Proteintech; Cat#10470-1-AP; RRID: AB_2276405), Rabbit polyclonal anti-ARFGAP1 (Proteintech; Cat#13571-1-AP; RRID: AB_2058621), Rabbit polyclonal anti-CCT8 (Sigma-Aldrich; Cat#HPA021051; RRID: AB_1846205), Rabbit polyclonal anti-TP53BP2 (Sigma-Aldrich; Cat#HPA021603; RRID: AB_1844384), Rabbit polyclonal anti-MIB1 (Sigma; Cat#M5948; RRID: AB_1841007), Mouse anti-HSP60 (Translab; Cat#H99020), Mouse monoclonal anti-ubiquitin (ZTA10, kind gift from Simona Polo), Rat monoclonal anti-CETN2 (BioLegend; Cat#698602; RRID: AB_2715794), Goat polyclonal anti-Mouse HRP-linked (Jackson ImmunoResearch; Cat#115-035-062; RRID: AB_2338504), Goat polyclonal anti-Rabbit HRP-linked (Jackson ImmunoResearch; Cat#111-035-045; RRID: AB_2337938), Goat polyclonal anti-Rabbit Alexa Fluor 488-linked (Invitrogen; Cat#A-11034; RRID: AB_2576217), Goat polyclonal anti-Mouse Alexa Fluor 488-linked (Invitrogen; Cat#A-11029; RRID: AB_2534088), Donkey polyclonal anti-Mouse Alexa Fluor 647-linked (Invitrogen; Cat#A-31571; RRID: AB_162542), Goat polyclonal anti-Rabbit Alexa Fluor 647-linked (Invitrogen; Cat#A-21244; RRID: AB_2535812), Goat polyclonal anti-Rat Alexa Fluor 647-linked (Invitrogen; Cat#A-21247; RRID: AB_141778), Streptavidin Alexa Fluor 594-linked (Jackson ImmunoResearch; Cat#016-290-084; RRID: AB_2337247). |
| Validation | All the antibodies described above are validated on the websites of the manufacturer or via RRIDs: Goat polyclonal anti-biotin HRP-linked (Cell Signaling; Cat#7075S): validated for WB in the website [https://www.cellsignal.com/products/secondary-antibodies/anti-biotin-hrp-linked-antibody/7075].Used for WB in this study. Rabbit polyclonal anti-BirA (SinoBiological; Cat#11582-T16): validated in the website [https://www.sinobiological.com/antibodies/bira-11582-rp01] and in this study using overexpression approaches. Used for IF and WB in this study. Mouse monoclonal anti-BirA (Novus Biologicals; Cat#NBP2-59939): validated for WB and IF in the website [https://www.novusbio.com/products/bira-antibody-5b11c3-3_nbp2-59939] and in this study using overexpression approaches. Used in this study for WB. Rabbit polyclonal anti-AviTag (GenScript; Cat#A00674): validated in the website [https://www.genscript.com/antibody/A00674-Avi_tag_Antibody_pAb_Rabbit.html] and in this study using overexpression approaches. Used for WB and IF in this study. We also validated that Rabbit polyclonal anti-AviTag (GenScript; Cat#A00674) antibody recognizes bioGEF tag by WB and IF. Mouse monoclonal anti-His tag (Genescript; Cat#A00186S): validated for WB in the website [https://www.genscript.com/antibody/A00186S-THE_His_Tag_Antibody_mAb_Mouse.html]. Used for WB in this study. Rabbit polyclonal anti-PCM1 (Cell Signaling; Cat#5213S; RRID: AB_10556960): several applications validated and published on the website [https://www.cellsignal.com/products/primary-antibodies/pcm-1-g2000-antibody/5213], including WB. Used for WB in this study. Mouse monoclonal anti-GAPDH clone 1E6D9 (Proteintech; Cat#60004-1-Ig): several applications validated and published in the website [https://www.ptglab.com/products/GAPDH-Antibody-60004-1-Ig.htm] including WB. Used for WB in this study. Rabbit monoclonal anti-alpha-Actinin clone D6F6 (Cell Signaling Cat#6487S): validated and published in the website [https://www.cellsignal.com/products/primary-antibodies/a-actinin-d6f6-xp-rabbit-mab/6487] for WB and IF. Used in this study for WB. Rabbit polyclonal anti-USP9X (Proteintech; Cat#55054-1-AP): several applications validated and published in the website [https://www.ptglab.com/ |

products/USP9X-Antibody-55054-1-AP.htm] including WB. Used for WB in this study. Rabbit polyclonal anti-CEP131 (Proteintech; Cat#25735-1-AP): several applications validated and published in the website [https://www.ptglab.com/products/AZI1-Antibody-25735-1-AP.htm] including WB. Used for WB in this study. Mouse monoclonal anti-SUMO2/3 (Proteintech; Cat#67154-1-Ig): several applications validated and published in the website [https://www.ptglab.com/products/SUMO2-3-Antibody-67154-1-Ig.htm] including WB. Used for WB in this study. Rabbit polyclonal anti-PML (Proteintech; Cat#21041-1-AP): several applications validated and published in the website [https://www.ptglab.com/products/PML-Antibody-21041-1-AP.htm] including WB and IF. Used for WB and IF in this study. Rabbit polyclonal anti-ROCK1 (Proteintech; Cat#21850-1-AP): several applications validated and published in the website [https://www.ptglab.com/products/ROCK1-Antibody-21850-1-AP.htm] including WB. Used for WB in this study. Rabbit polyclonal anti-GIGYF2 (Proteintech; Cat#24790-1-AP): several applications validated and published in the website [https://www.ptglab.com/products/GIGYF2-Antibody-24790-1-AP.htm] including WB. Used for WB in this study. Rabbit polyclonal anti-CLINT1 (Proteintech; Cat#10470-1-AP): several applications validated and published in the website [https://www.ptglab.com/products/CLINT1-Antibody-10470-1-AP.htm] including WB. Used for WB in this study. Rabbit polyclonal anti-ARFGAP1 (Proteintech; Cat#13571-1-AP): several applications validated and published in the website [https://www.ptglab.com/products/ARFGAP1-Antibody-13571-1-AP.htm] including WB. Used for WB in this study. Rabbit polyclonal anti-MIB1 (Sigma; Cat#M5978): Validated in the website for several applications, including WB [https://www.sigmaaldrich.com/ES/es/product/sigma/m5948]. Used for WB in this study. Rabbit polyclonal anti-CCT8 (Sigma-Aldrich; Cat#HPA021051): several applications validated and published in the website [https://www.sigmaaldrich.com/ES/es/product/sigma/hpa021051] including WB. Used for WB in this study. Rabbit polyclonal anti-TP53BP2 (Sigma-Aldrich; Cat#HPA021603): validated and published in the website for immunohistochemistry [https://www.sigmaaldrich.com/ES/es/product/sigma/hpa021603]. Used for WB in this study. Mouse anti-HSP60 (Translab; Cat#H99020): validated and used for IF in this study. Mouse monoclonal anti-ubiquitin (ZTA10, kind gift from Simona Polo) was validated in [https://www.nature.com/articles/nsmb.2566] for WB. Used for WB in this study. Rat monoclonal anti-CETN2 (BioLegend; Cat#698602): validated and published in the website for immunohistochemistry [https://www.biolegend.com/en-us/products/purified-anti-centrin-2-caltractin-antibody-14768]. Used for IF in this study.

## Eukaryotic cell lines

Policy information about cell lines and Sex and Gender in Research

| | |
|---|---|
| Cell line source(s) | U2OS (ATCC; HTB-96; RRID: CVCL_0042)<br>HEK 293FT (Invitrogen; Cat#R70007; RRID: CVCL_6911) |
| Authentication | U2OS and HEK 293FT cells were used at low passage after purchase or acquisition.<br>All generated stable cell lines were regularly verified by WB. |
| Mycoplasma contamination | All the cell lines used in the study were negative for mycoplasma contamination |
| Commonly misidentified lines<br>(See ICLAC register) | No cell lines used are listed in the database of commonly misidentified cell lines. |
