# Supplementary material for: BioE3 identifies specific substrates of ubiquitin E3 ligases
Source: Nat Commun. 2023 Nov 23;14:7656. doi: 10.1038/s41467-023-43326-8 (PMC10667490; doi:10.1038/s41467-023-43326-8)

Fig. 2a

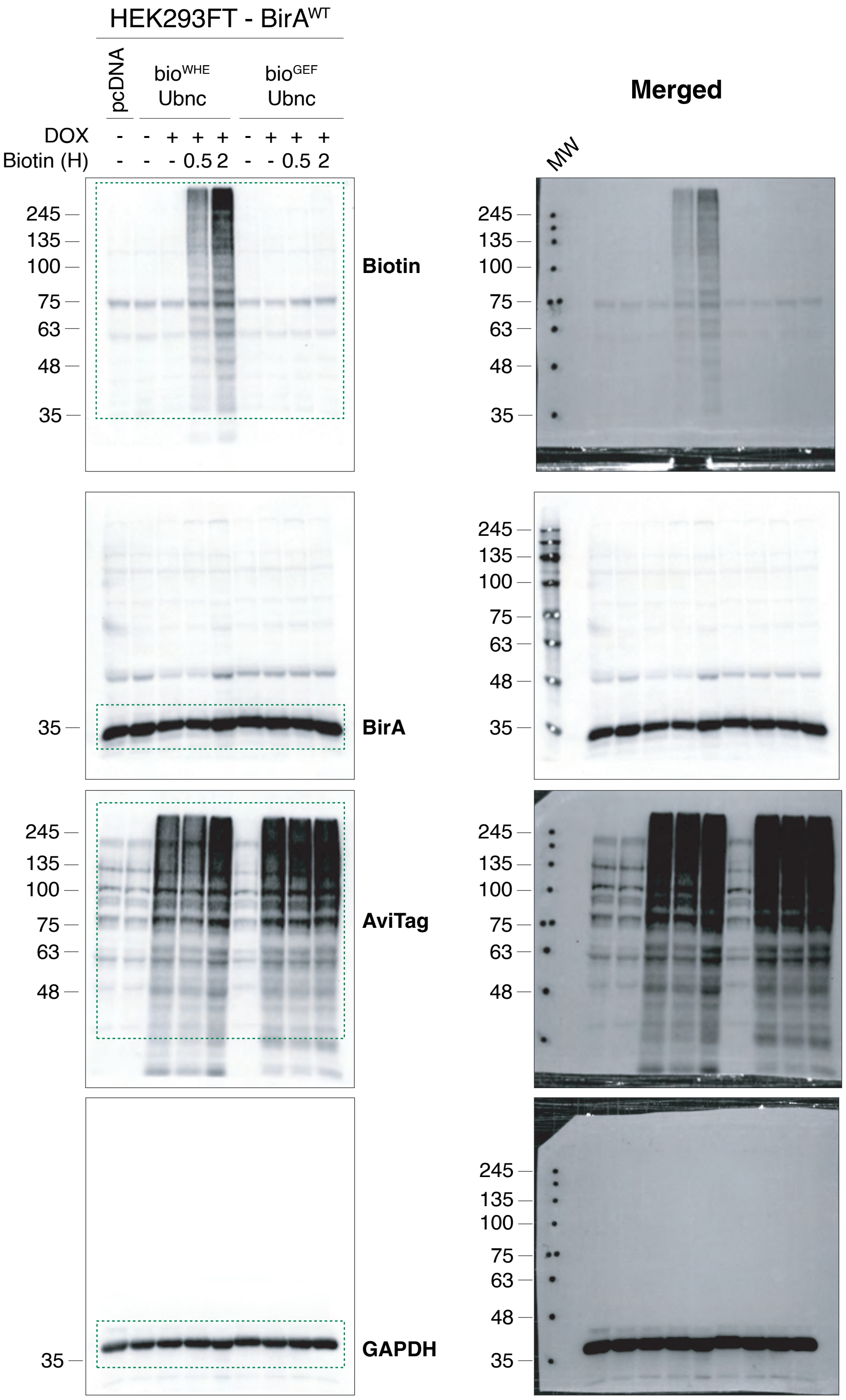
HEK293FT - BirA^WT
pcDNA
bio^WHE Ubnc
bio^GEF Ubnc
DOX - - + + + - + + +
Biotin (H) - - - 0.5 2 - - 0.5 2
Merged
MW
245
135
100
75
63
48
35
Biotin
BirA
AviTag
GAPDH

Fig. 2b (left panel)

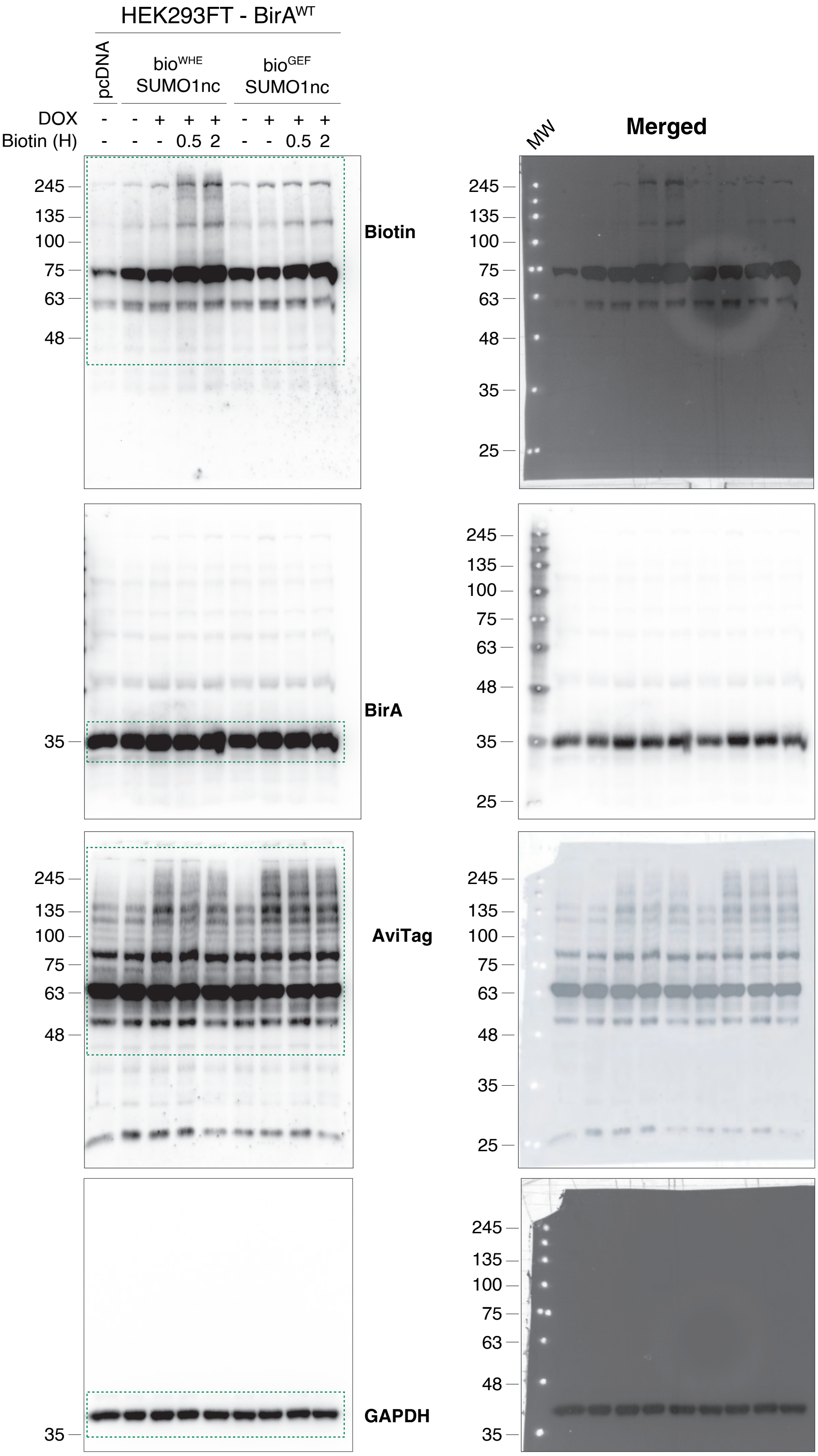

Fig. 2b (right panel)

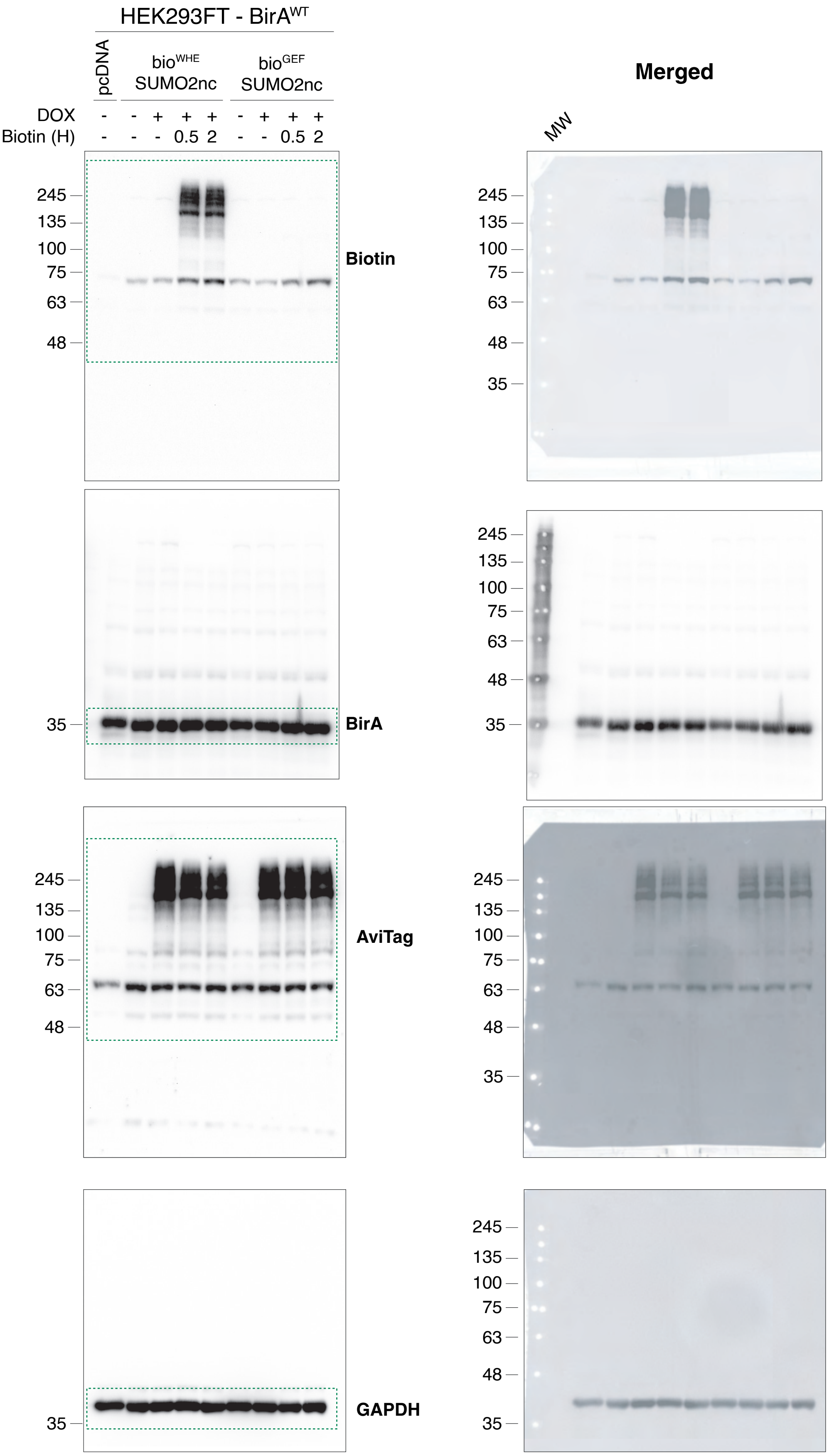

Fig. 3a

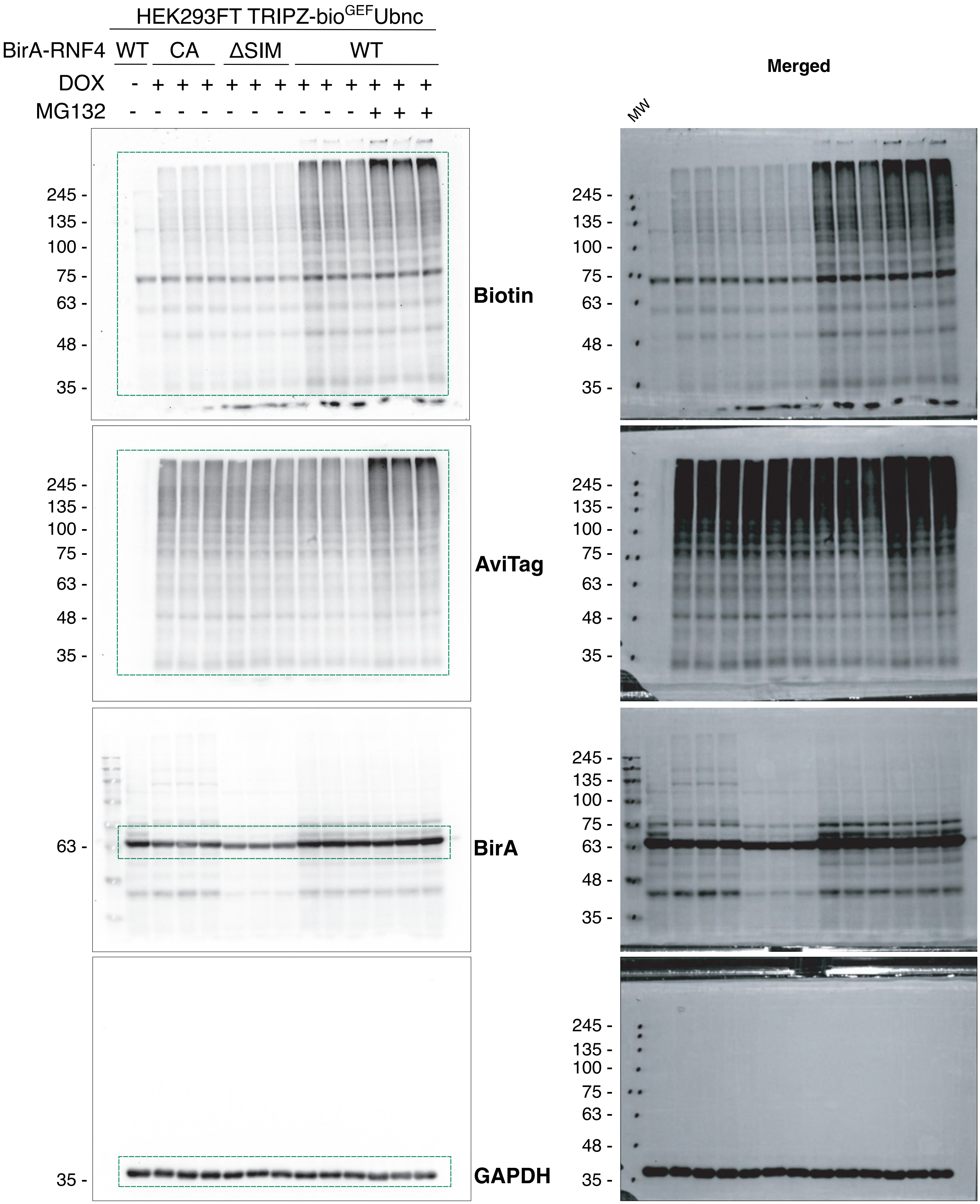
HEK293FT TRIPZ-bioGEFUbnc
BirA-RNF4 WT CA ΔSIM WT
DOX - + + + + + + + + + + + +
MG132 - - - - - - - - - - + + +
245 -
135 -
100 -
75 -
63 -
48 -
35 -
Biotin
AviTag
BirA
GAPDH
Merged
MW

Fig. 3c

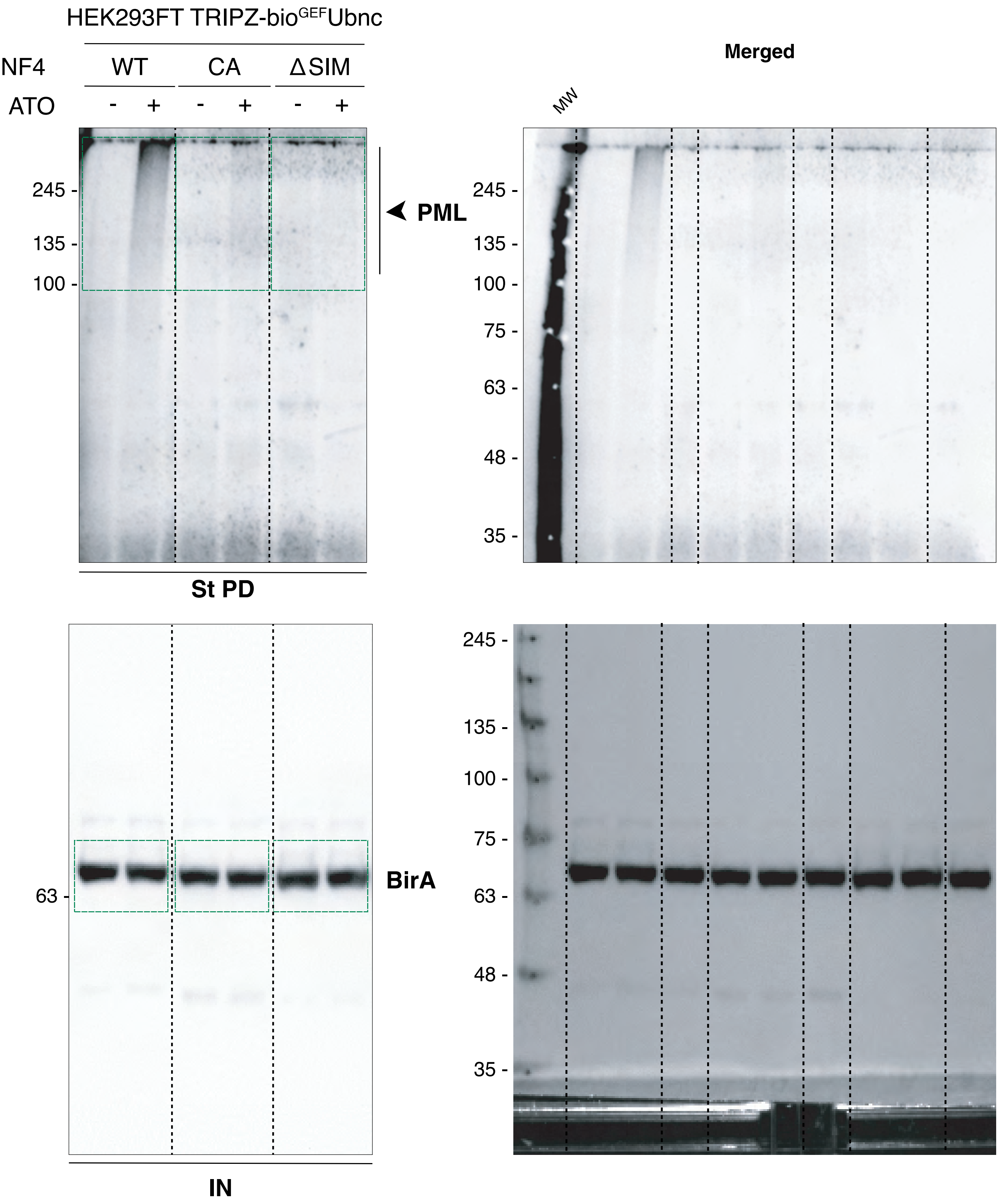
HEK293FT TRIPZ-bioGEFUbnc
BirA-RNF4
WT
CA
ΔSIM
ATO
- + - + - +
245 -
135 -
100 -
PML
St PD
63 -
BirA
IN
Merged
MW
245 -
135 -
100 -
75 -
63 -
48 -
35 -

Fig. 4c

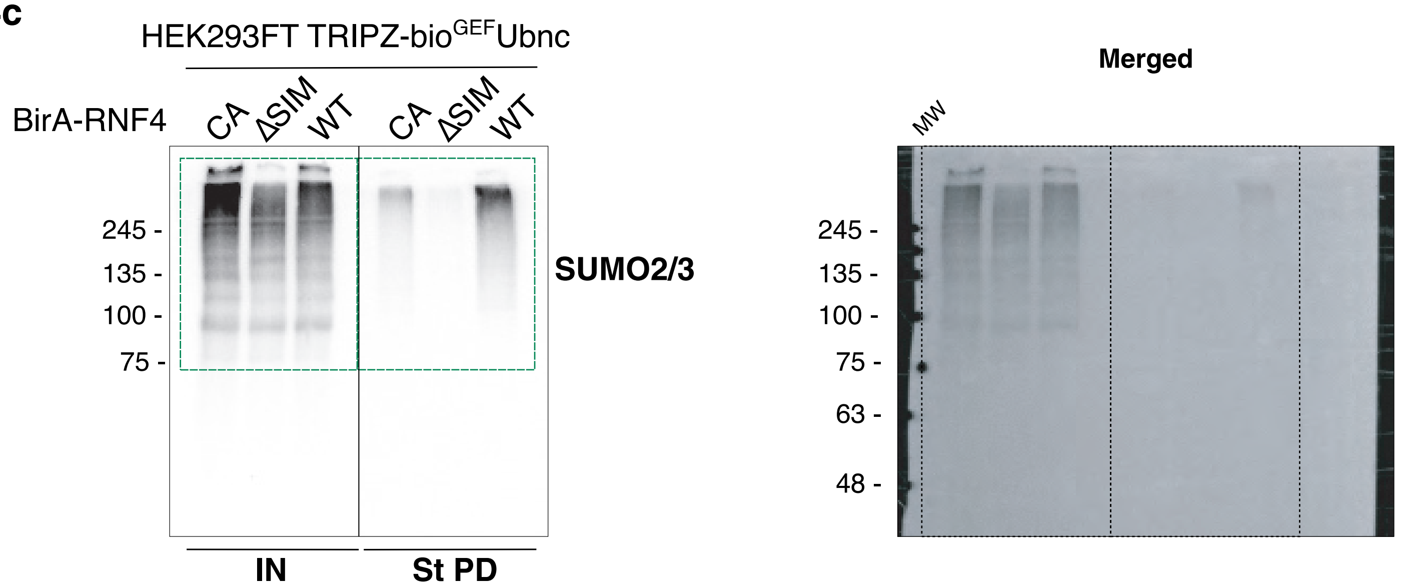
HEK293FT TRIPZ-bioGEFUbnc
BirA-RNF4
CA
ΔSIM
WT
CA
ΔSIM
WT
245 -
135 -
100 -
75 -
SUMO2/3
IN
St PD
Merged
MW
245 -
135 -
100 -
75 -
63 -
48 -

Fig. 6a

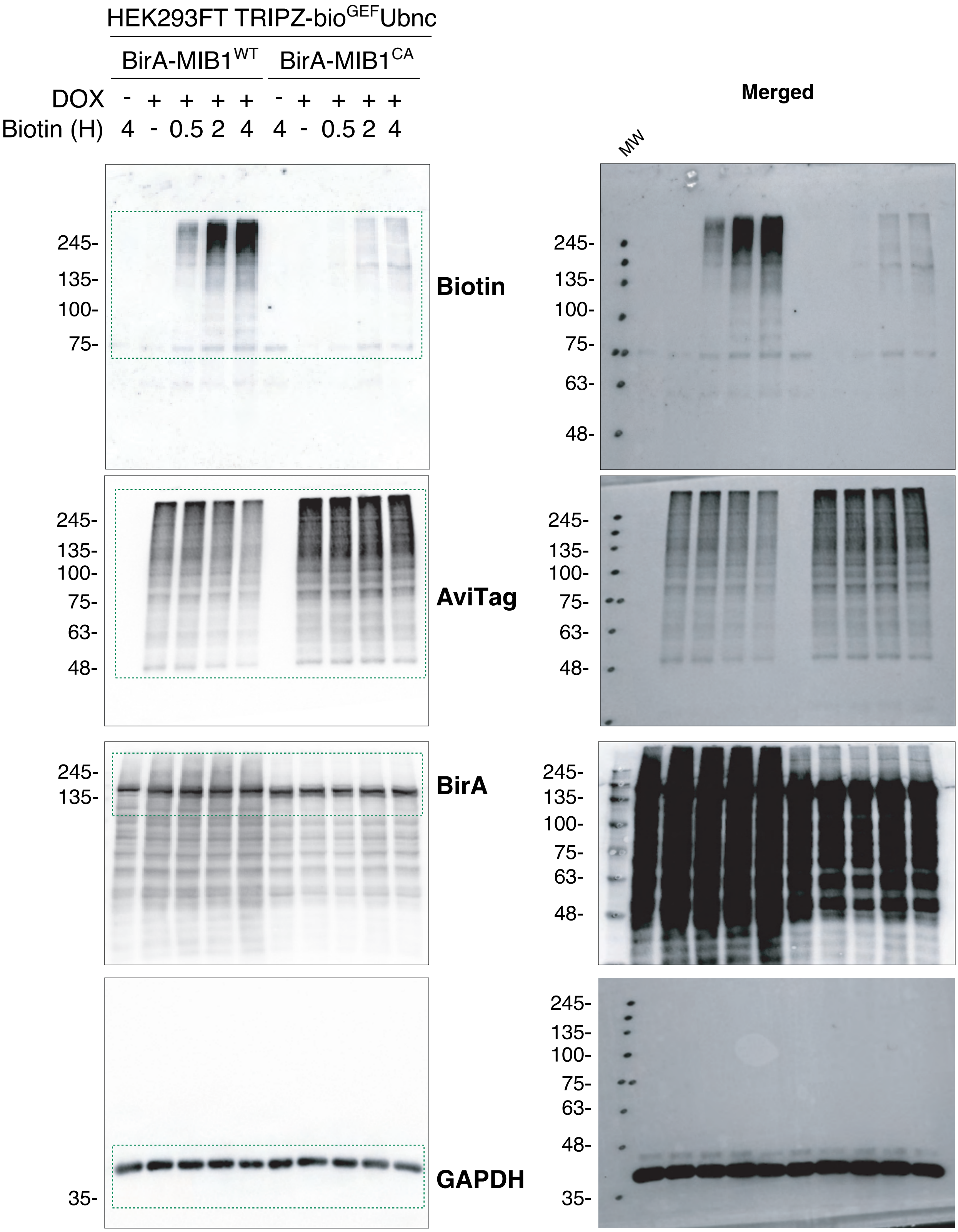

HEK293FT TRIPZ-bio^GEF Ubnc
BirA-MIB1^WT
BirA-MIB1^CA
DOX - + + + + - + + + +
Biotin (H) 4 - 0.5 2 4 4 - 0.5 2 4
Merged
MW
245-
135-
100-
75-
63-
48-
35-
Biotin
AviTag
BirA
GAPDH

Fig. 6d

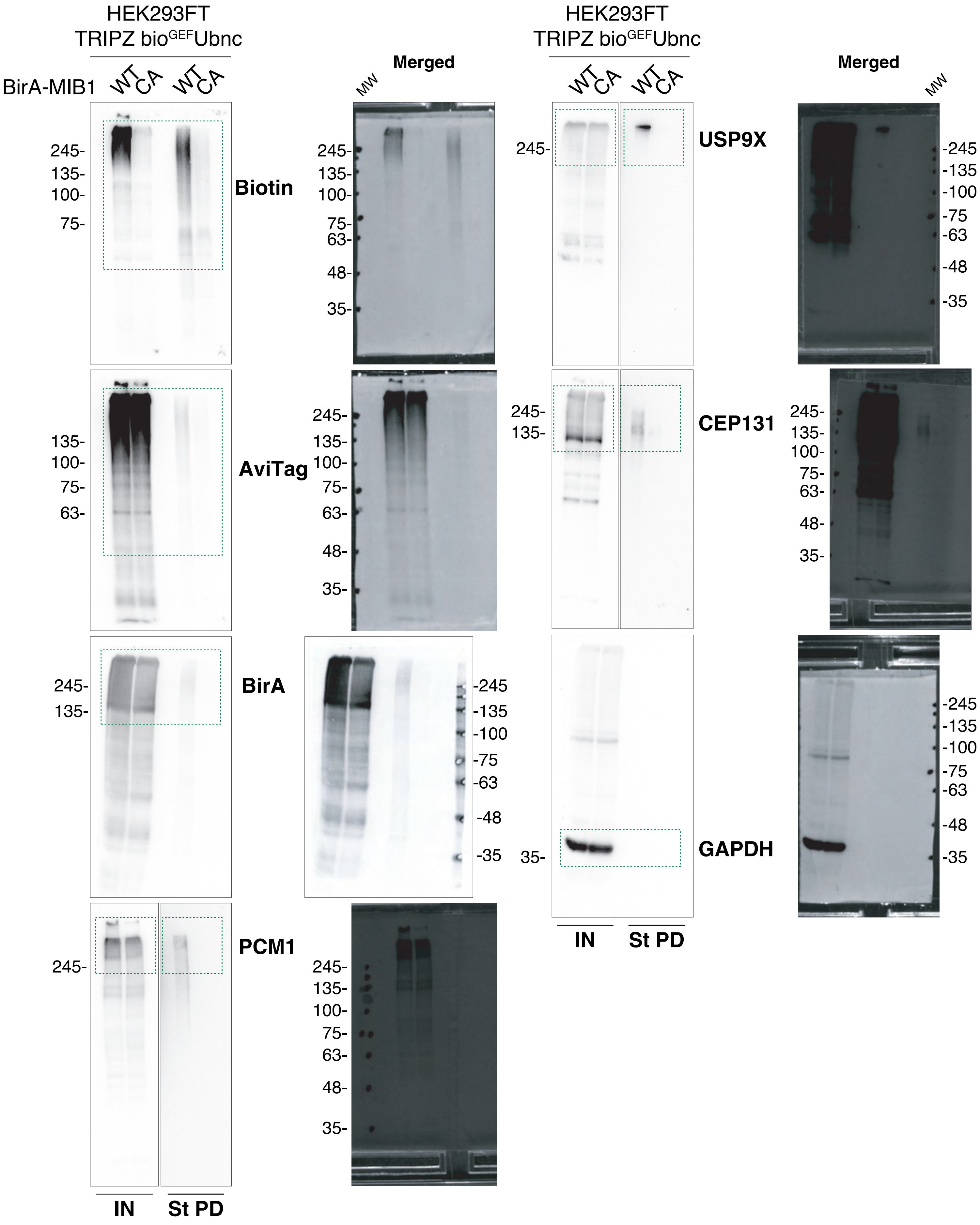

HEK293FT
TRIPZ bioGEFUbnc
BirA-MIB1
WT
CA
WT
CA
Merged
MW
Biotin
AviTag
BirA
PCM1
USP9X
CEP131
GAPDH
IN
St PD
245-
135-
100-
75-
63-
48-
35-

Fig. 7a

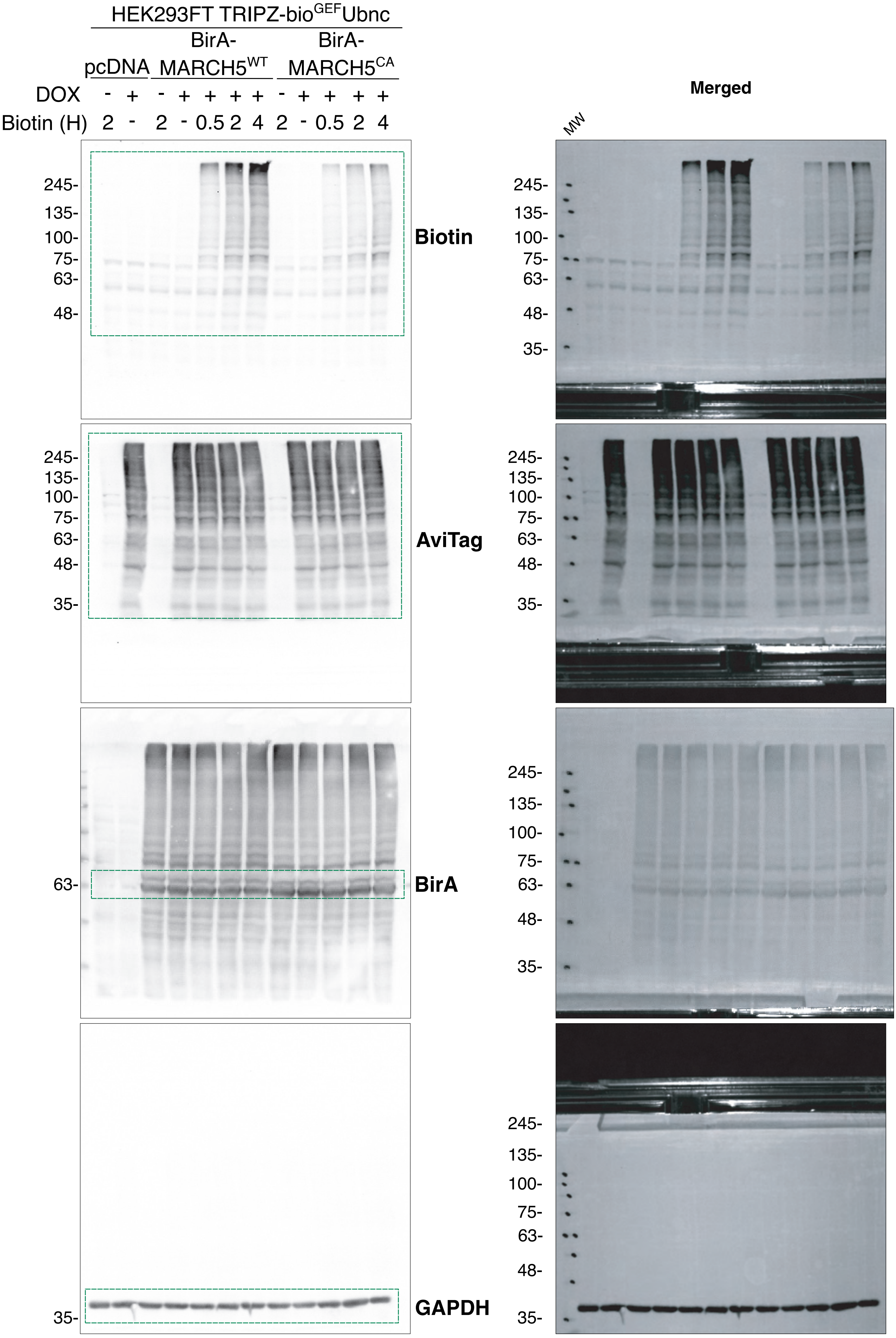

HEK293FT TRIPZ-bio GEF Ubnc
pcDNA
BirA-MARCH5 WT
BirA-MARCH5 CA
DOX - + - + + + + - + + + +
Biotin (H) 2 - 2 - 0.5 2 4 2 - 0.5 2 4
Merged
MW
245-
135-
100-
75-
63-
48-
35-
Biotin
AviTag
BirA
GAPDH

Fig. 7c

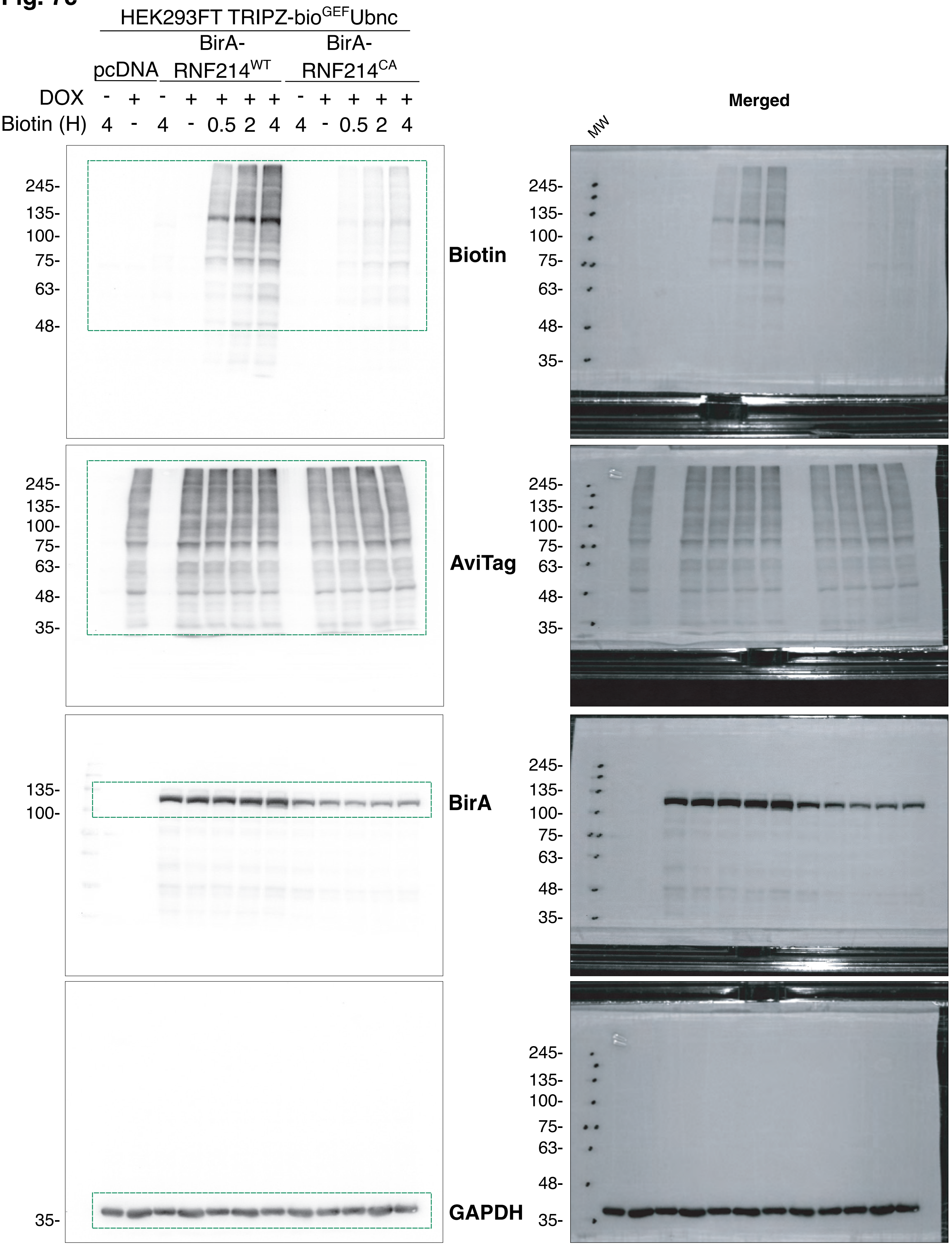
HEK293FT TRIPZ-bioGEFUbnc
pcDNA
BirA-RNF214WT
BirA-RNF214CA
DOX - + - + + + + - + + + +
Biotin (H) 4 - 4 - 0.5 2 4 4 - 0.5 2 4
Merged
MW
245-
135-
100-
75-
63-
48-
35-
Biotin
AviTag
BirA
GAPDH

Fig. 7f

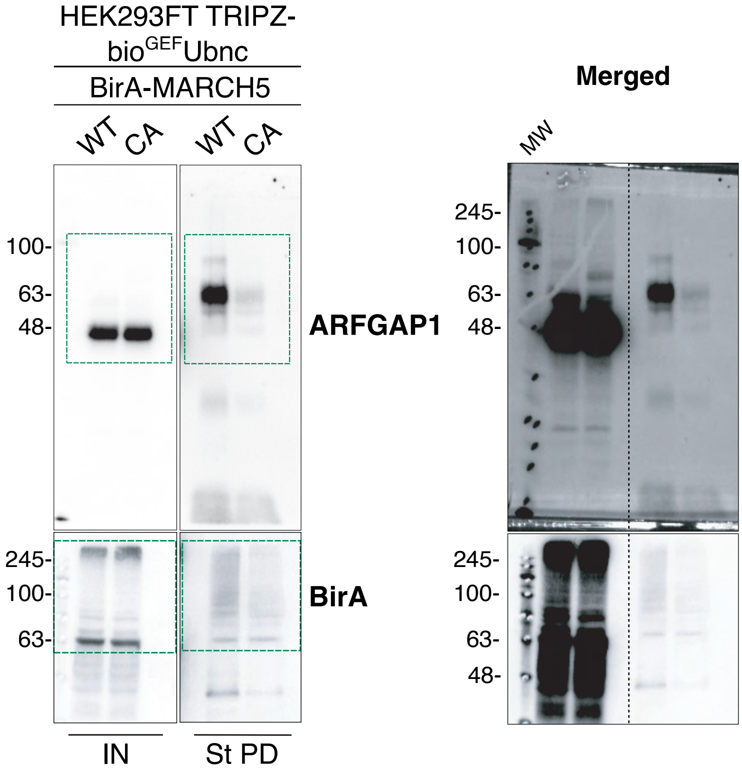

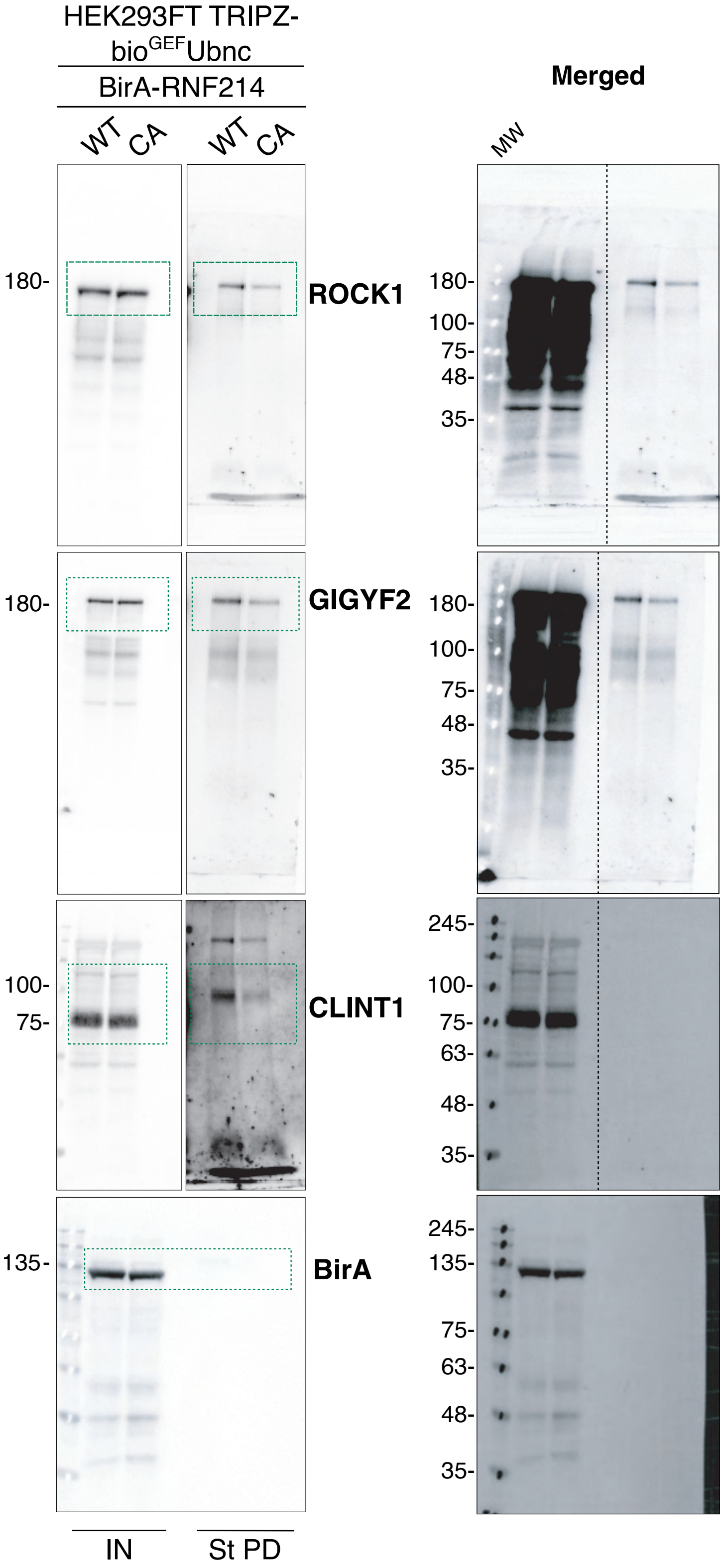

Fig. 8a

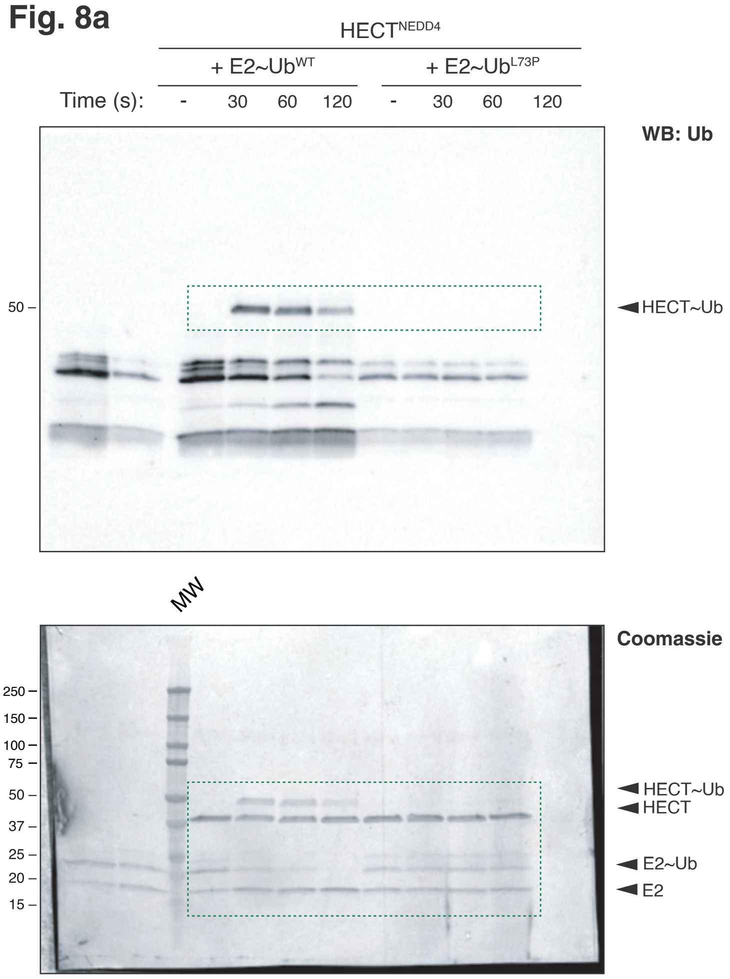

Fig. 8b

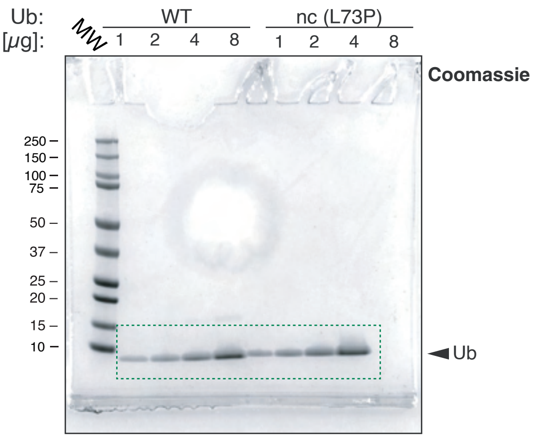

Fig. 8c

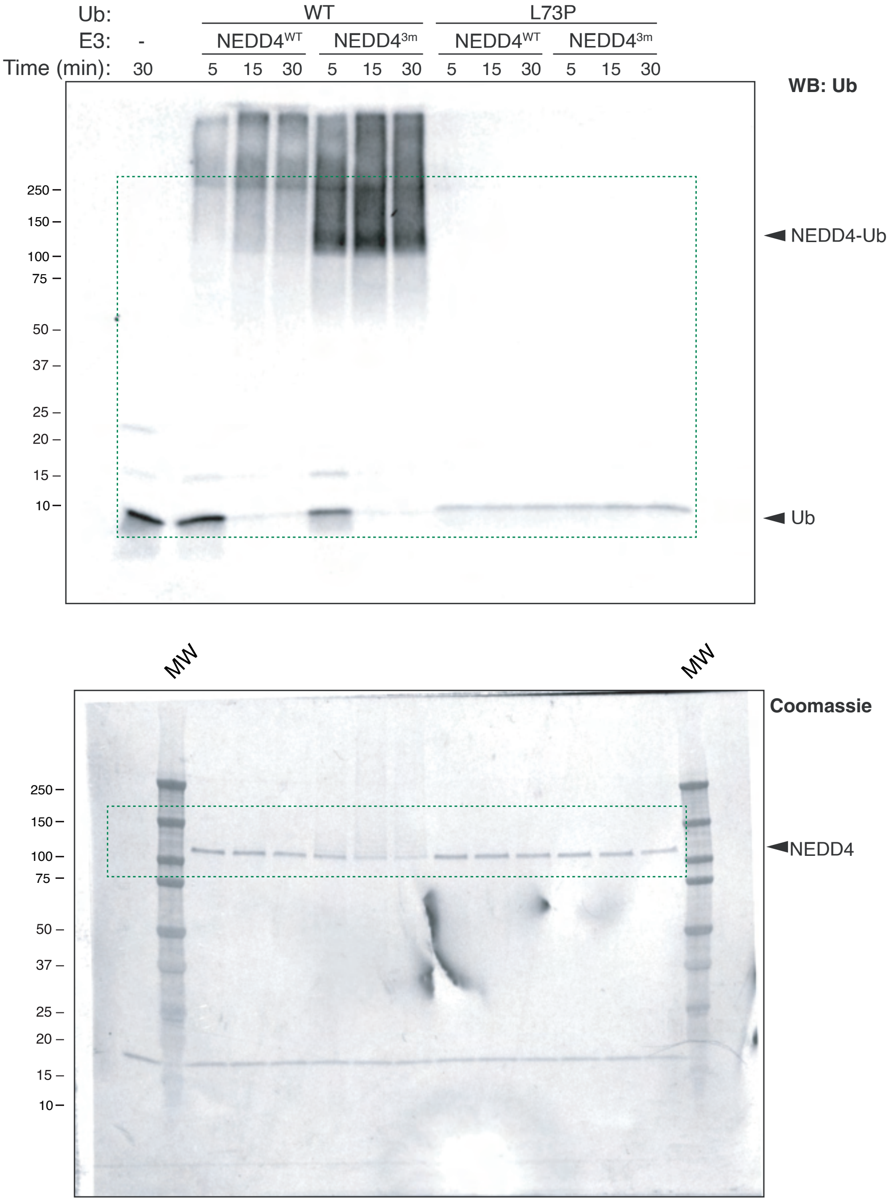
Ub:
WT
L73P
E3:
-
NEDD4WT
NEDD43m
NEDD4WT
NEDD43m
Time (min):
30
5 15 30 5 15 30 5 15 30 5 15 30
WB: Ub
250
150
100
75
50
37
25
20
15
10
NEDD4-Ub
Ub
MW
MW
Coomassie
250
150
100
75
50
37
25
20
15
10
NEDD4

Fig. 8d

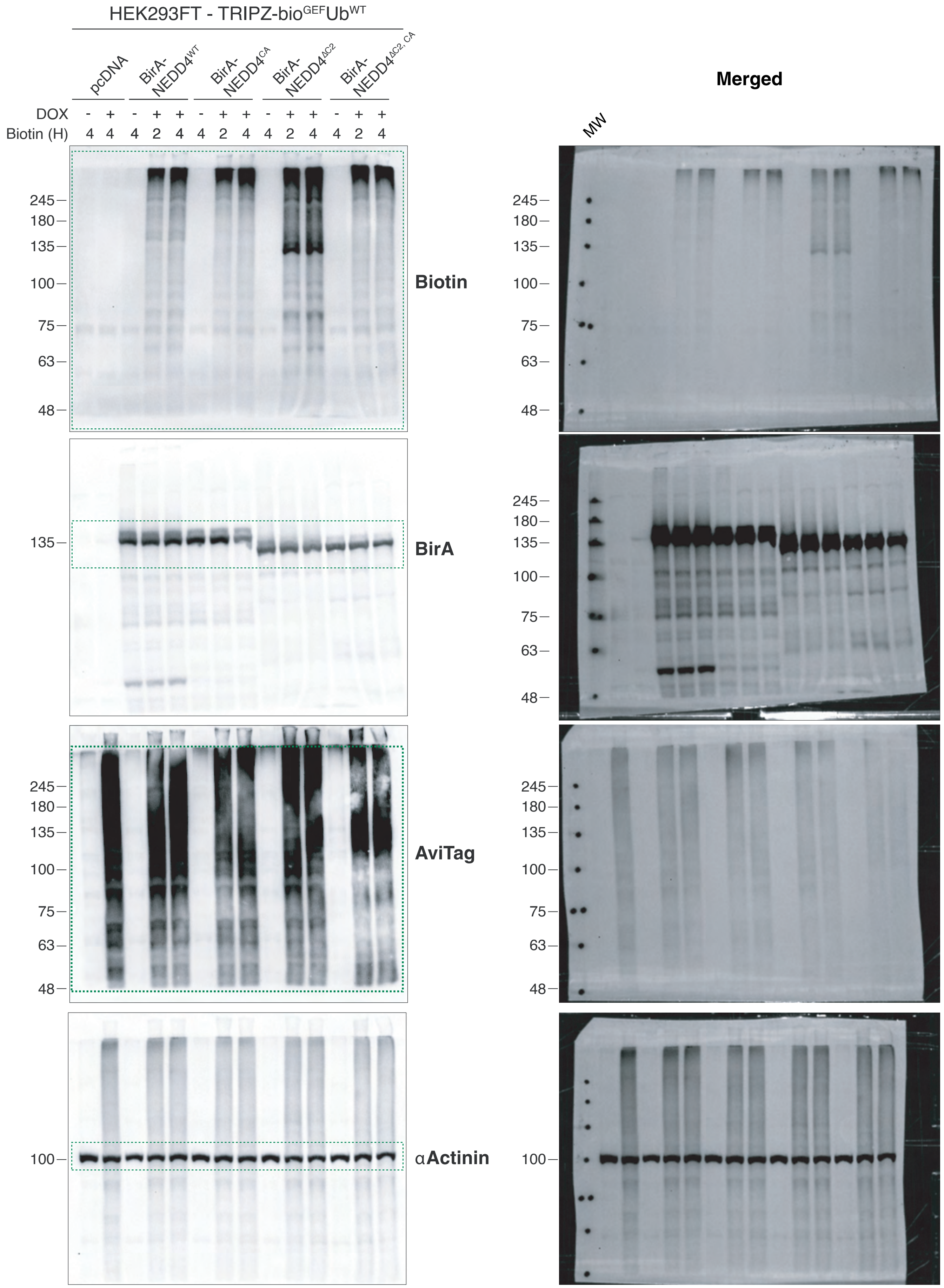
HEK293FT - TRIPZ-bio^GEF Ub^WT
pcDNA
BirA-NEDD4^WT
BirA-NEDD4^CA
BirA-NEDD4^ΔC2
BirA-NEDD4^ΔC2, CA
DOX - + - + + - + + - + + - + +
Biotin (H) 4 4 4 2 4 4 2 4 4 2 4 4 2 4
245
180
135
100
75
63
48
Biotin
BirA
AviTag
αActinin
Merged
MW

Fig. 8e

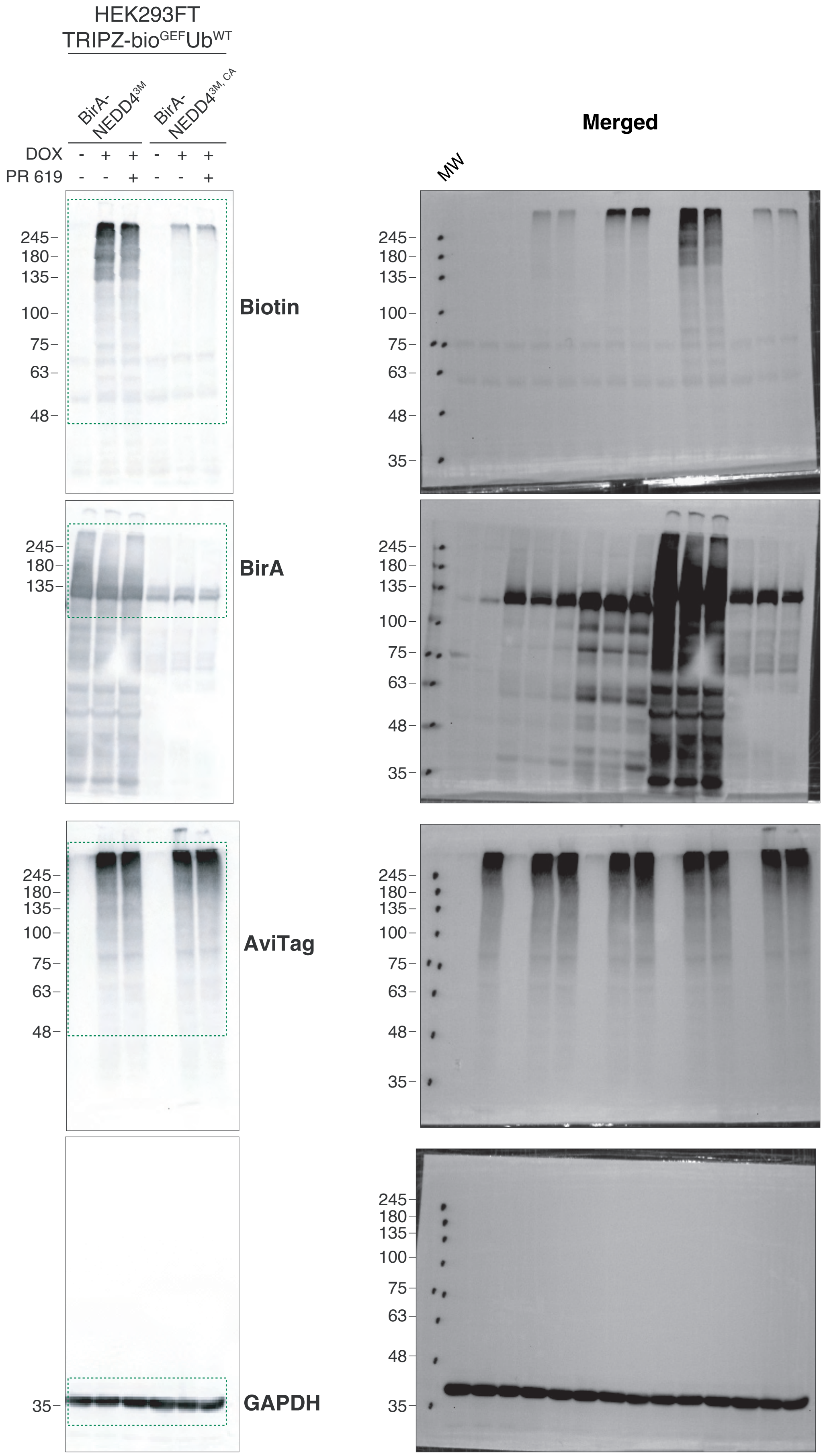

HEK293FT
TRIPZ-bio^{GEF}Ub^{WT}
BirA-NEDD4^{3M}
BirA-NEDD4$^{3M, CA}$
DOX - + + - + +
PR 619 - - + - - +
Merged
MW
245
180
135
100
75
63
48
35
Biotin
BirA
AviTag
GAPDH

Fig. 8h

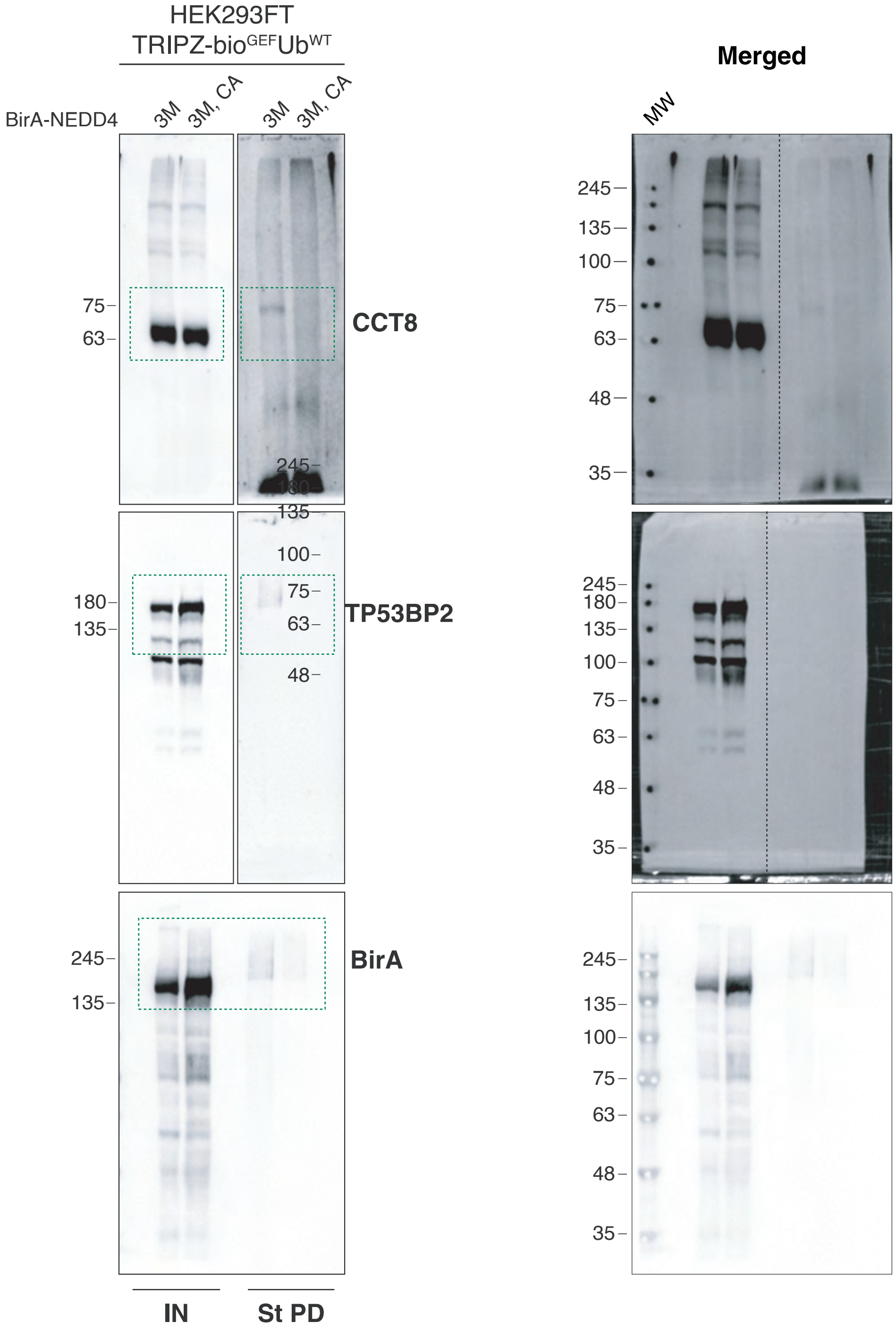
HEK293FT
TRIPZ-bio^GEF Ub^WT
Merged
BirA-NEDD4
3M
3M, CA
MW
CCT8
TP53BP2
BirA
IN
St PD
245
180
135
100
75
63
48
35

Supplementary Fig. 1a

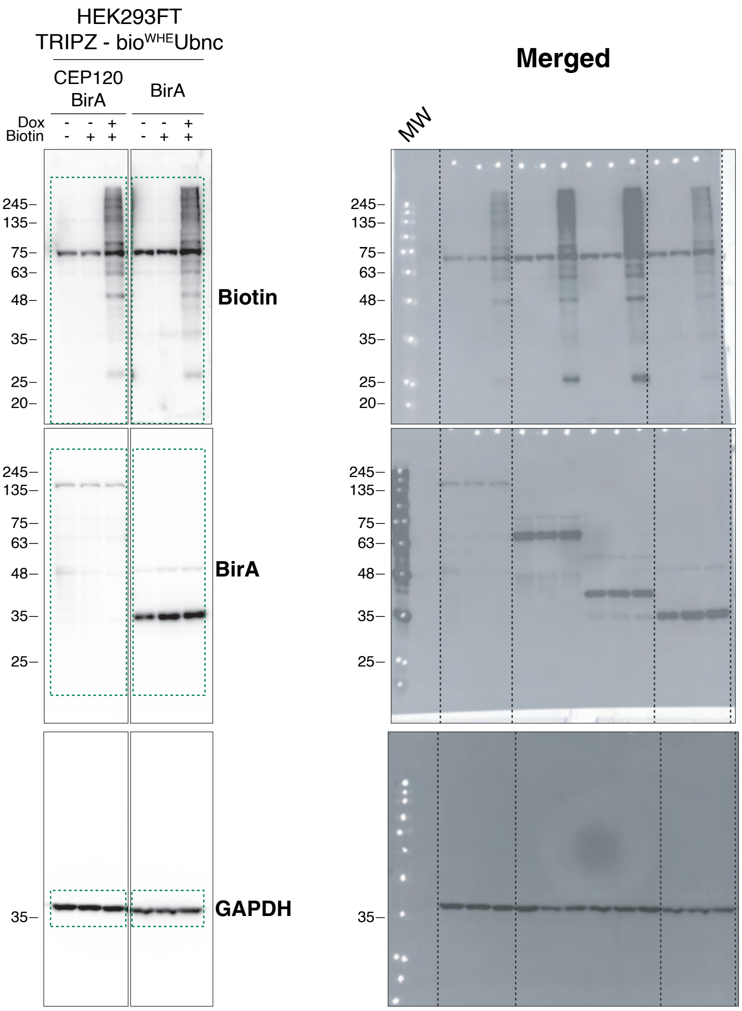

Supplementary Fig. 2a (left panel)

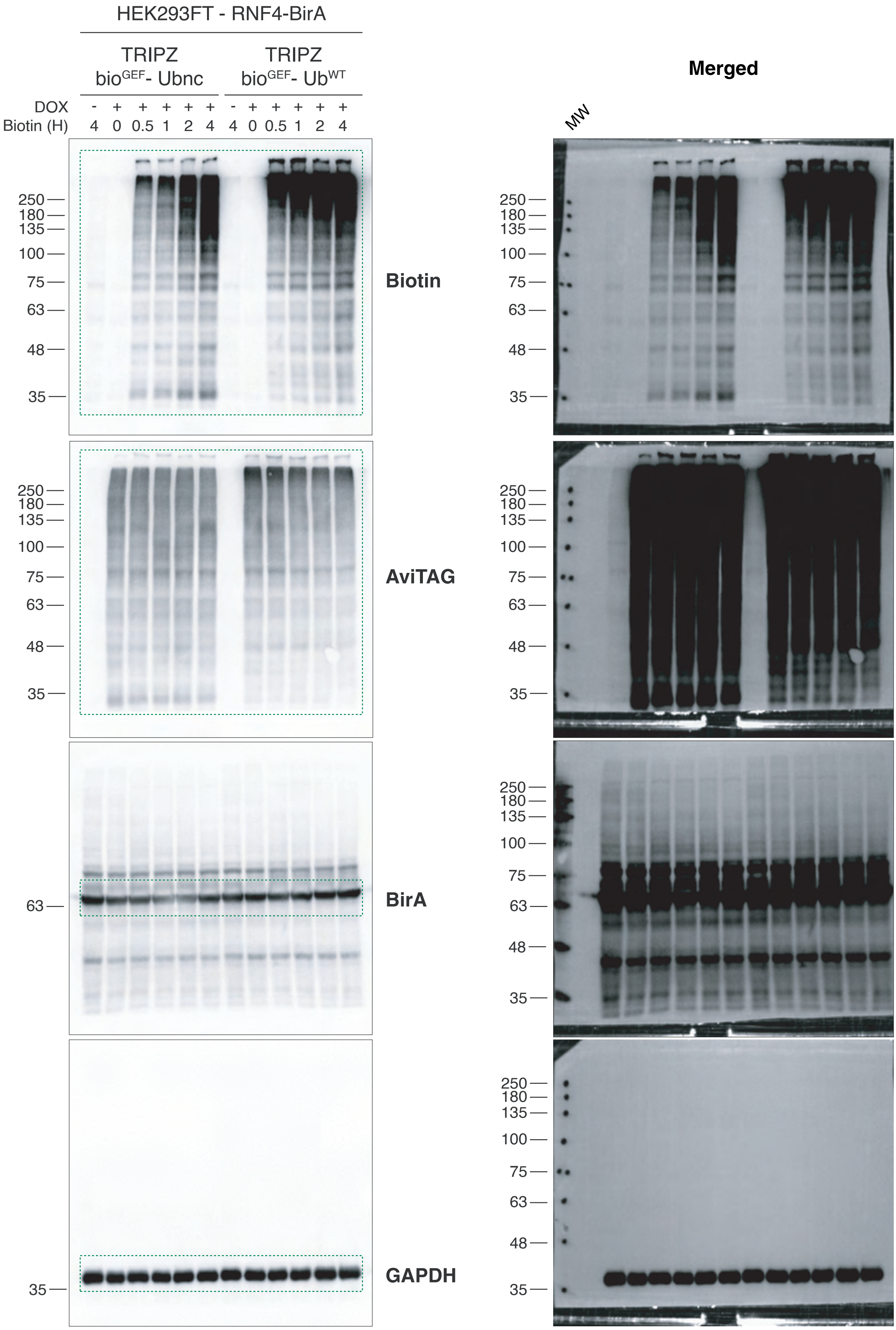

Supplementary Fig. 2a (right panel)

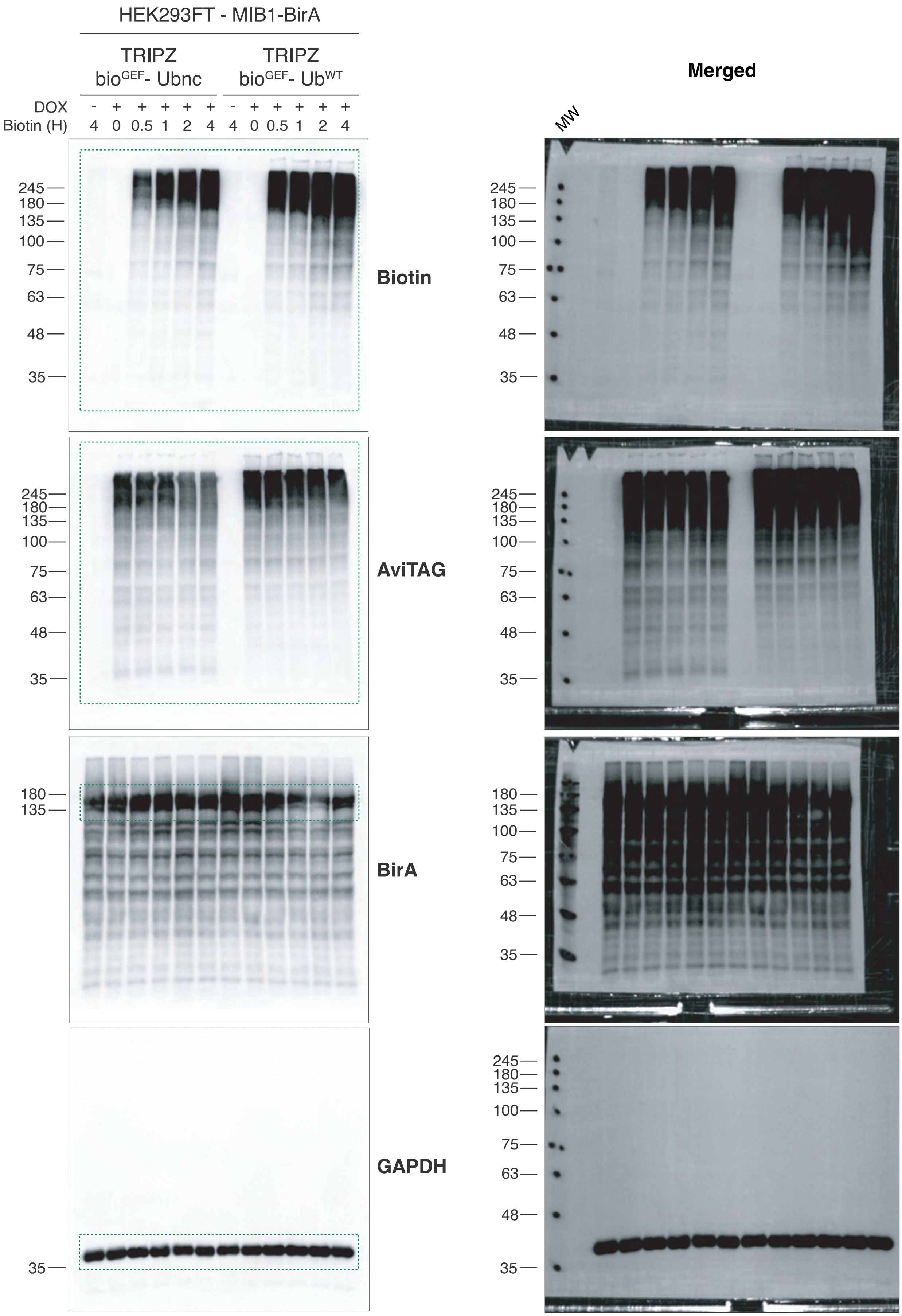

Supplementary Fig. 3 (left panel)

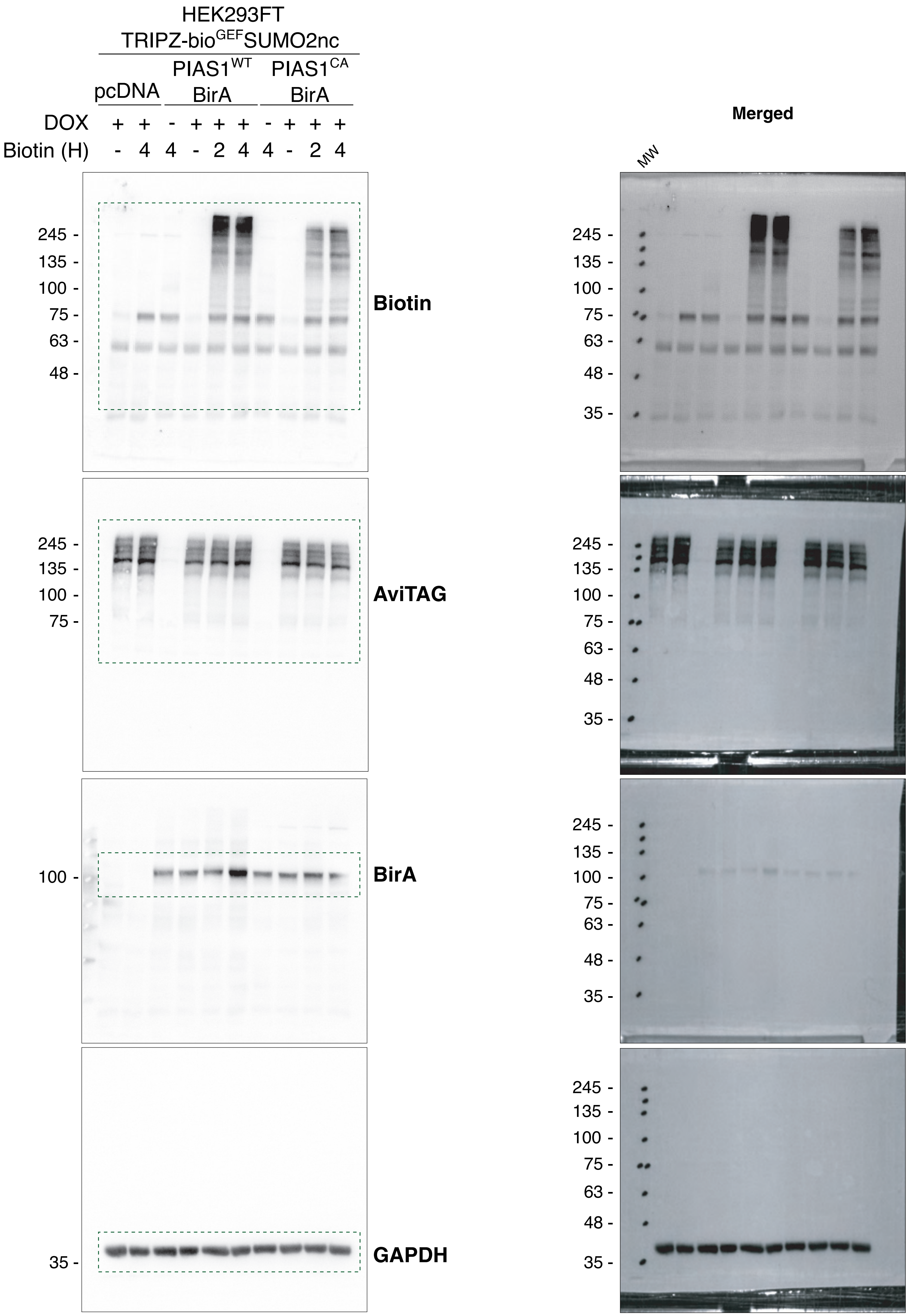

Supplementary Fig. 3 (right panel)

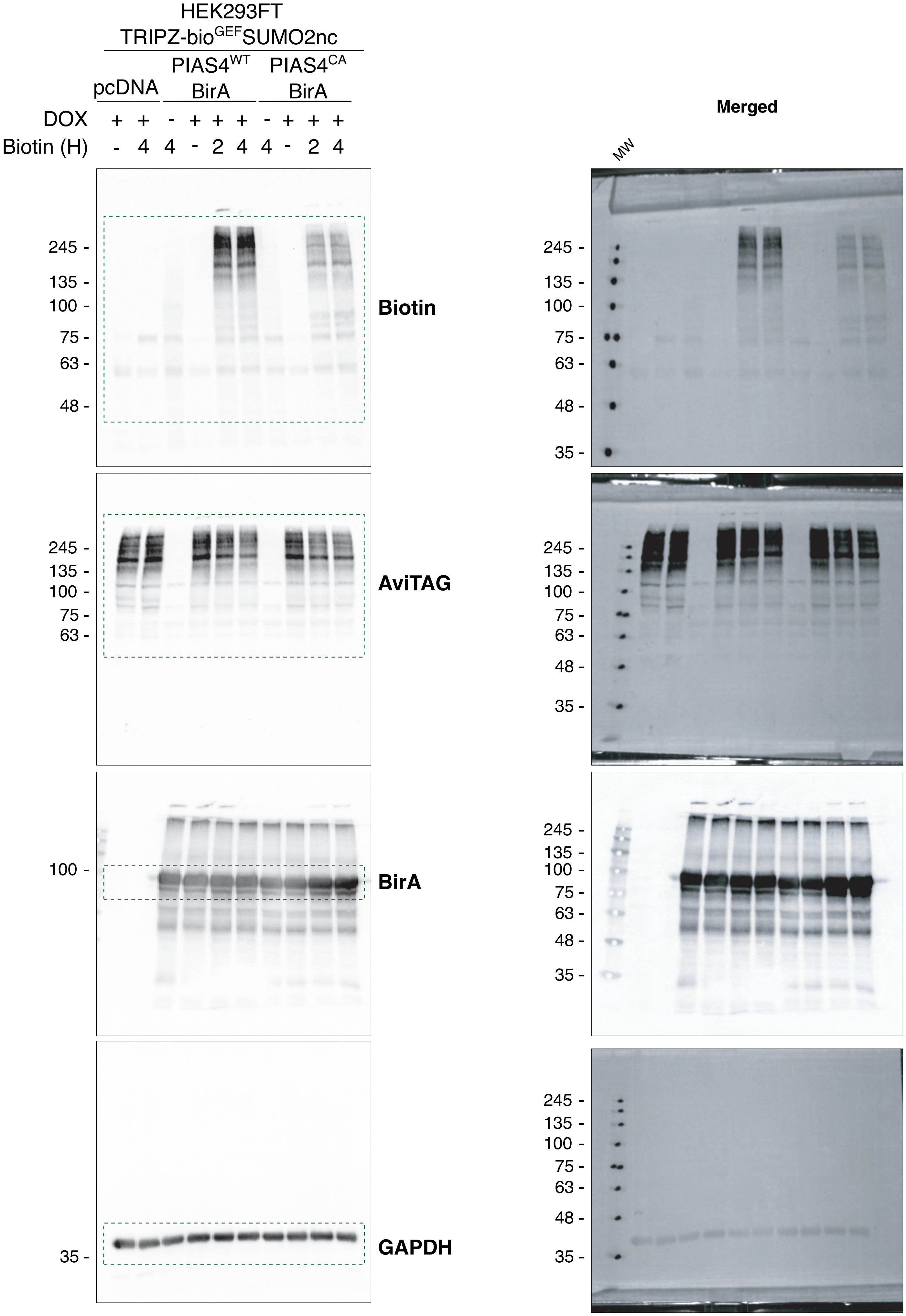

Supplementary Fig. 4

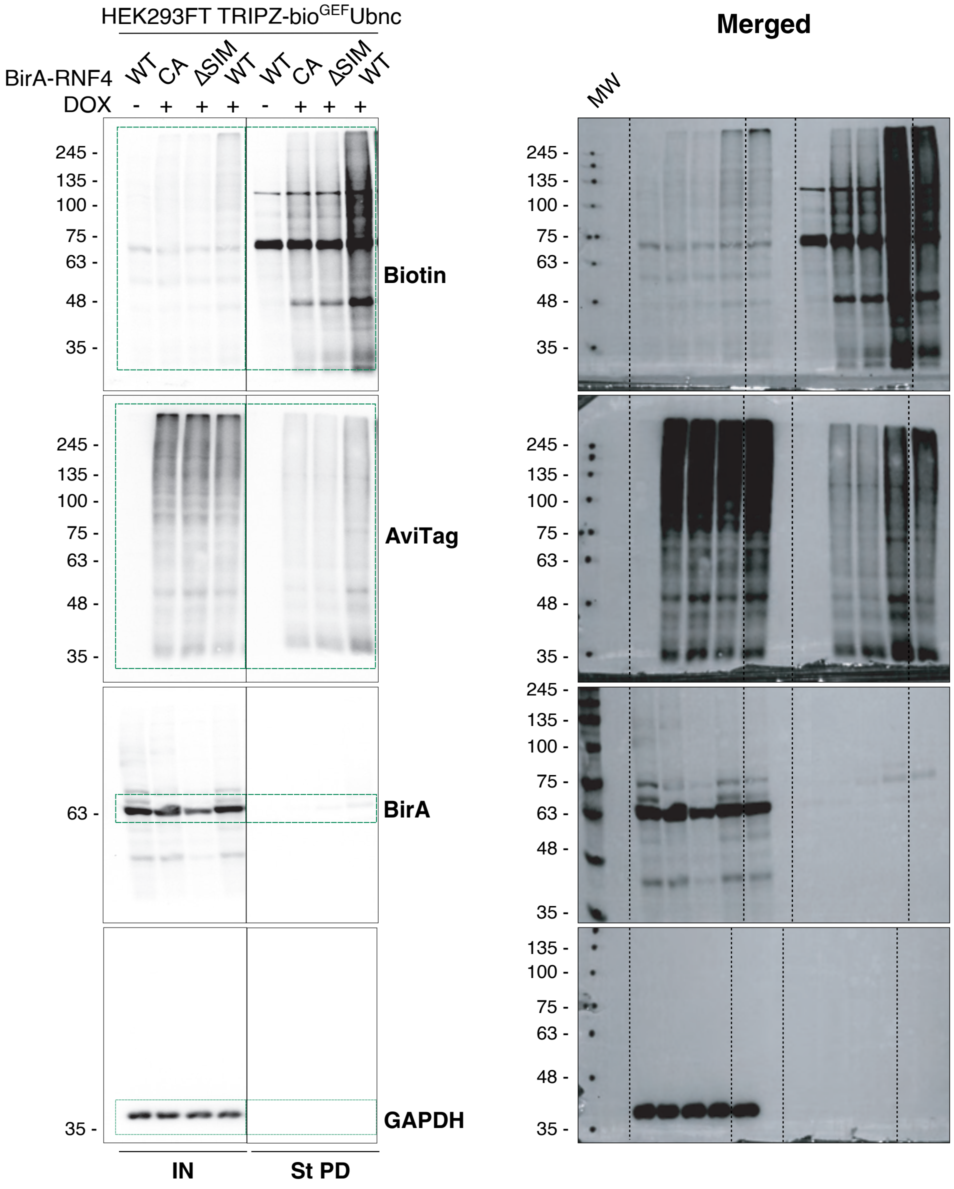

Supplementary Fig. 10a

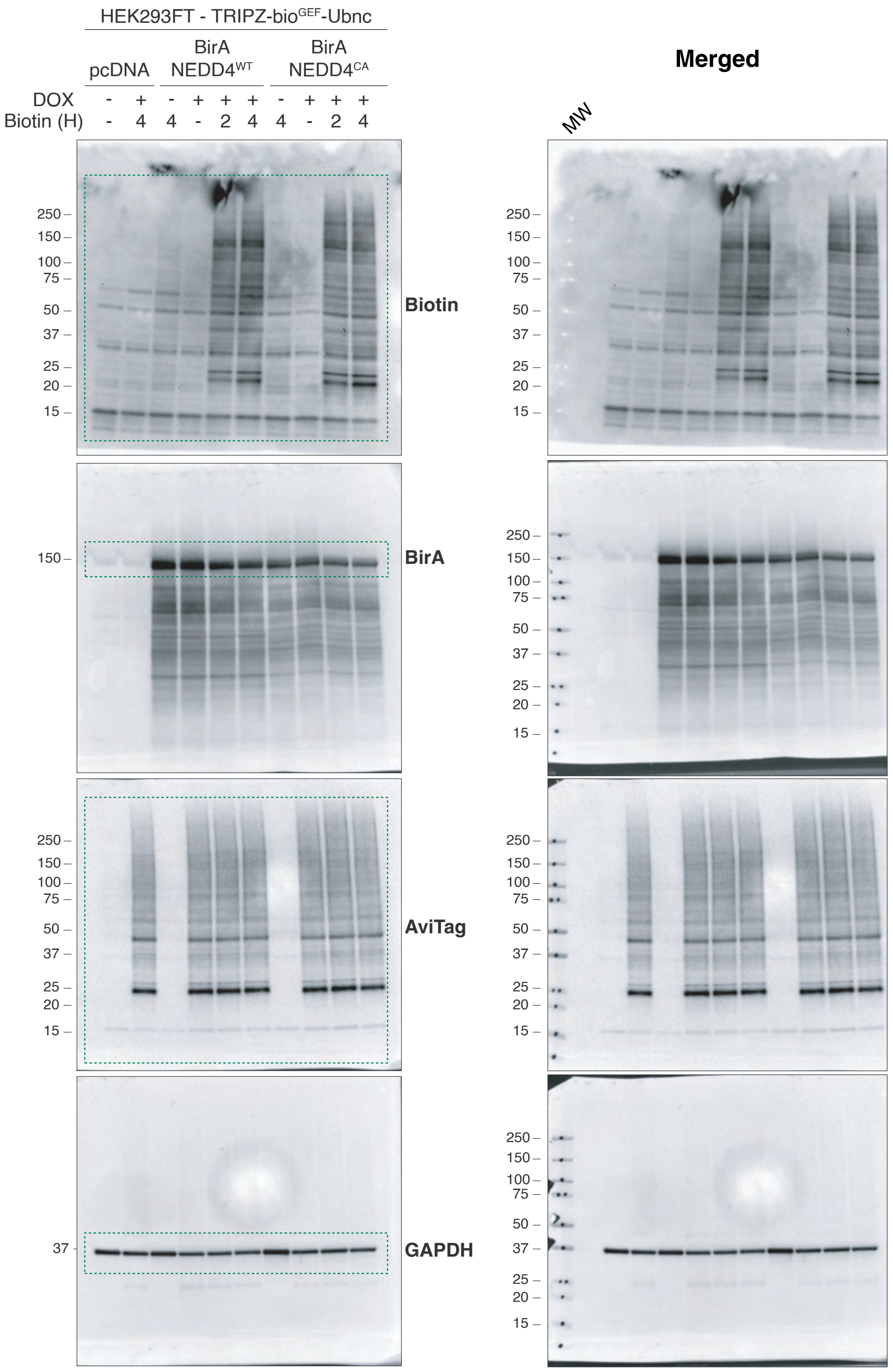

Supplementary Fig. 10c

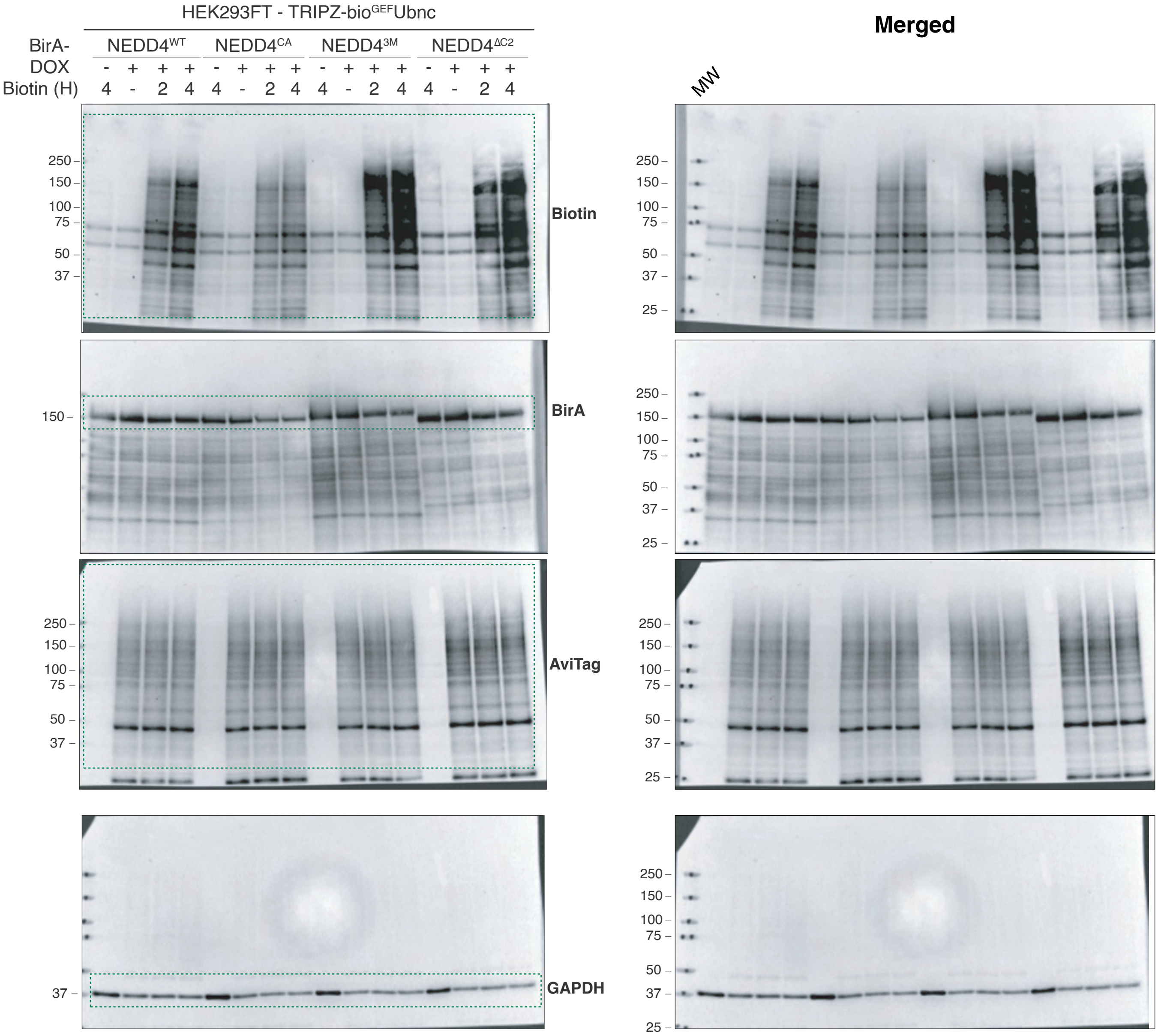

Supplementary Fig. 10e

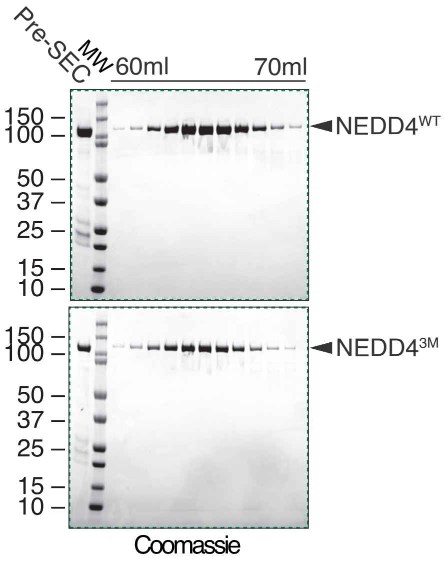

Supplementary Fig. 11a

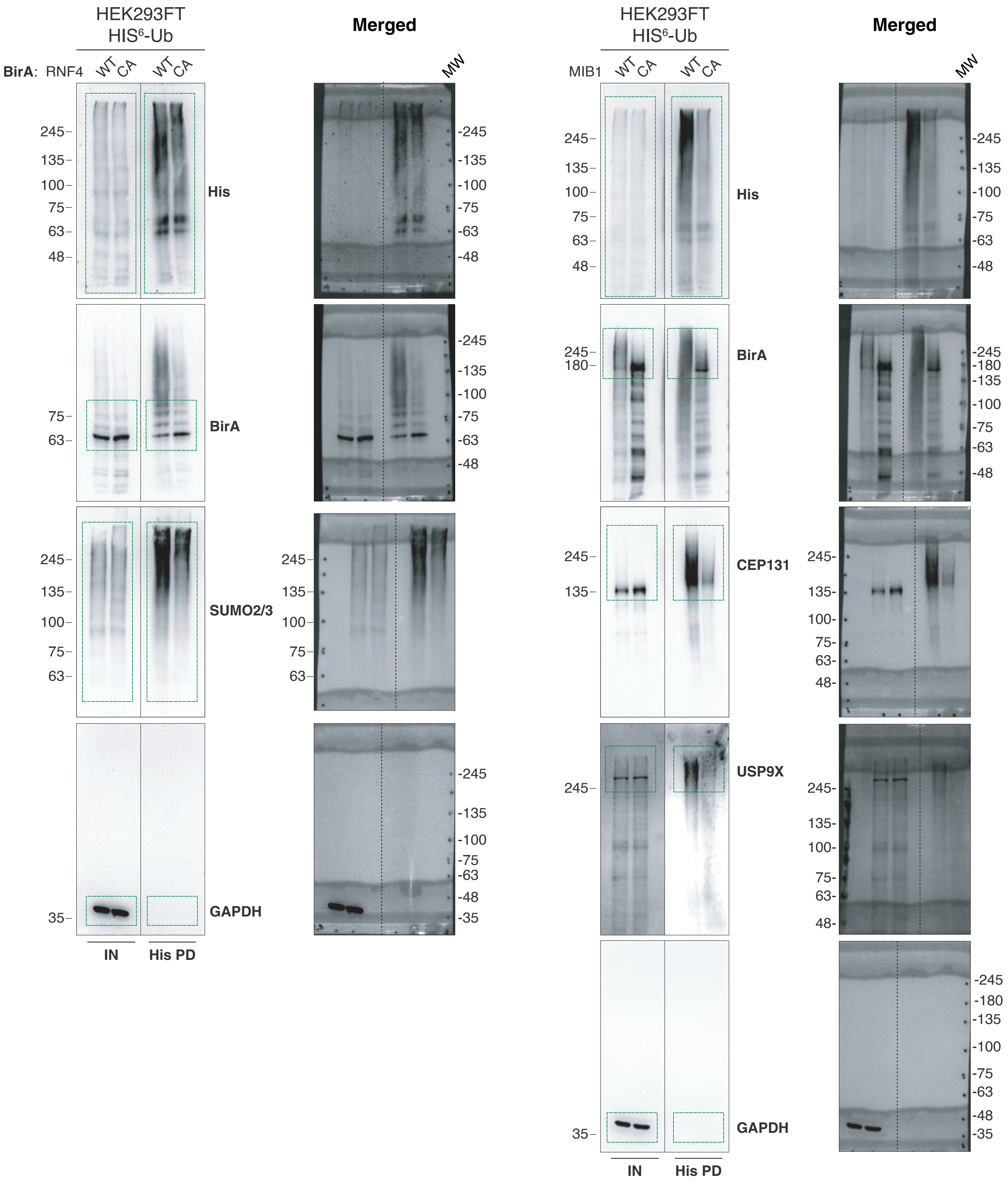

Supplementary Fig. 11a

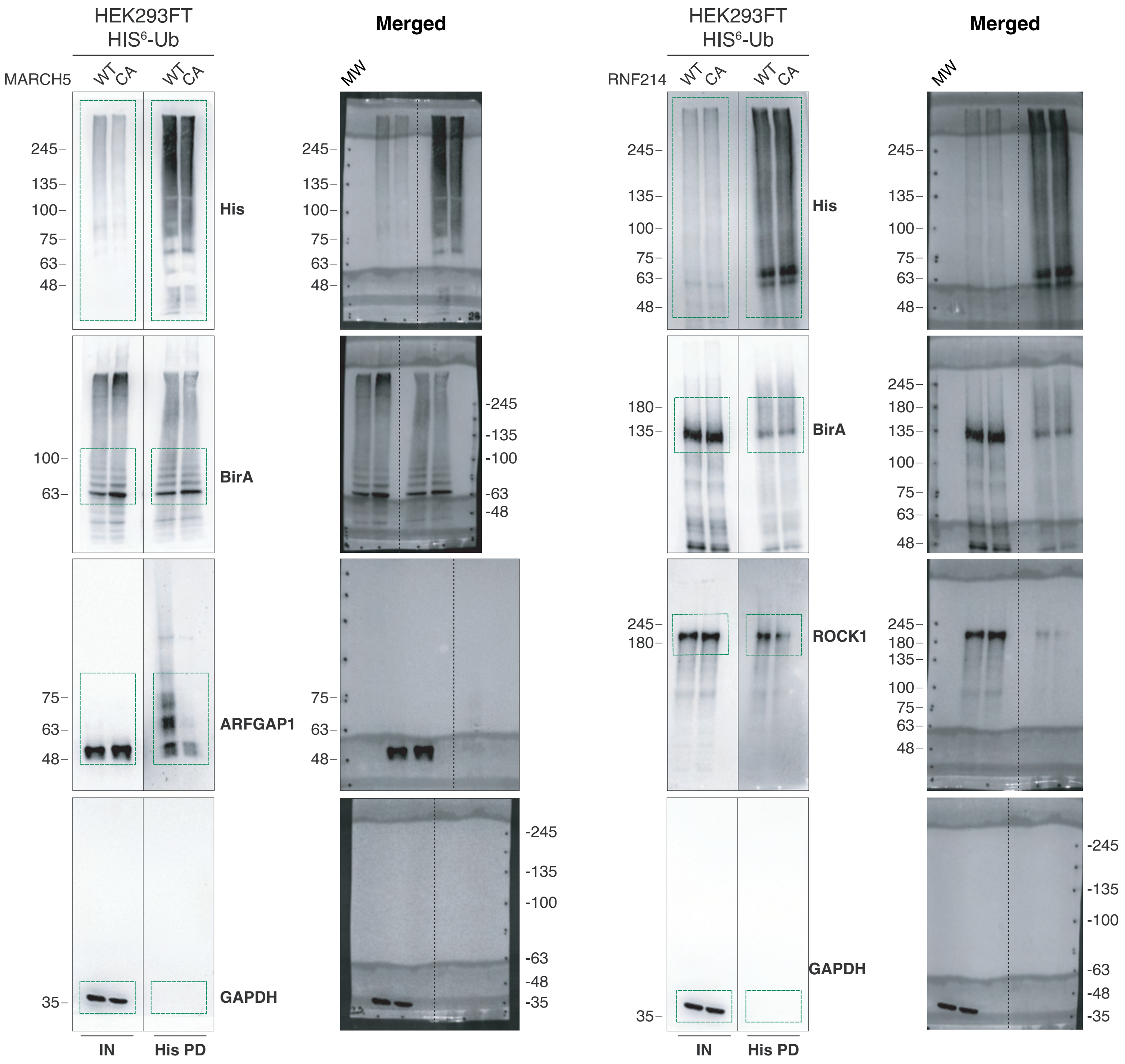

Supplementary Fig. 11a

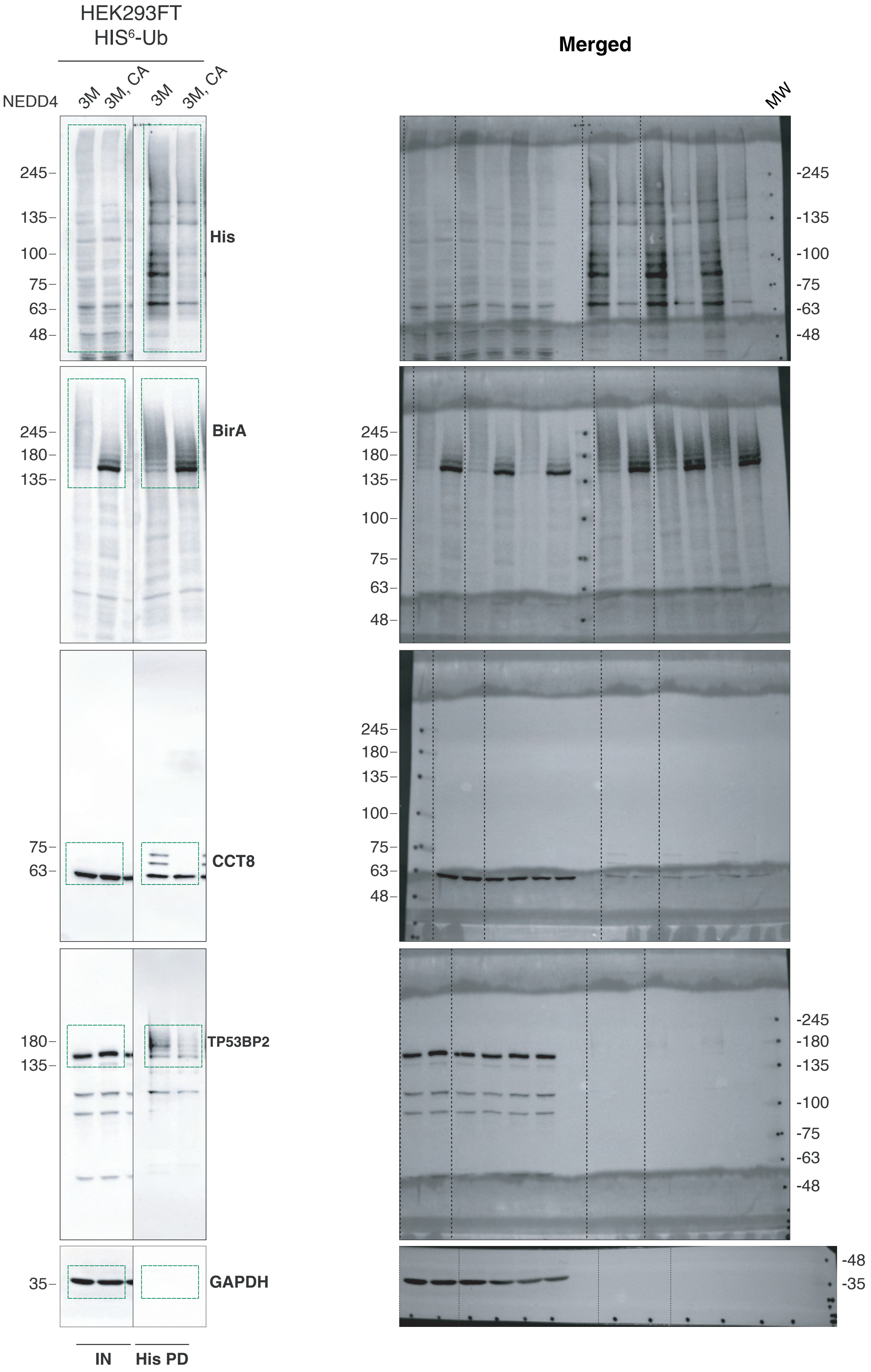

Supplementary Fig. 11b

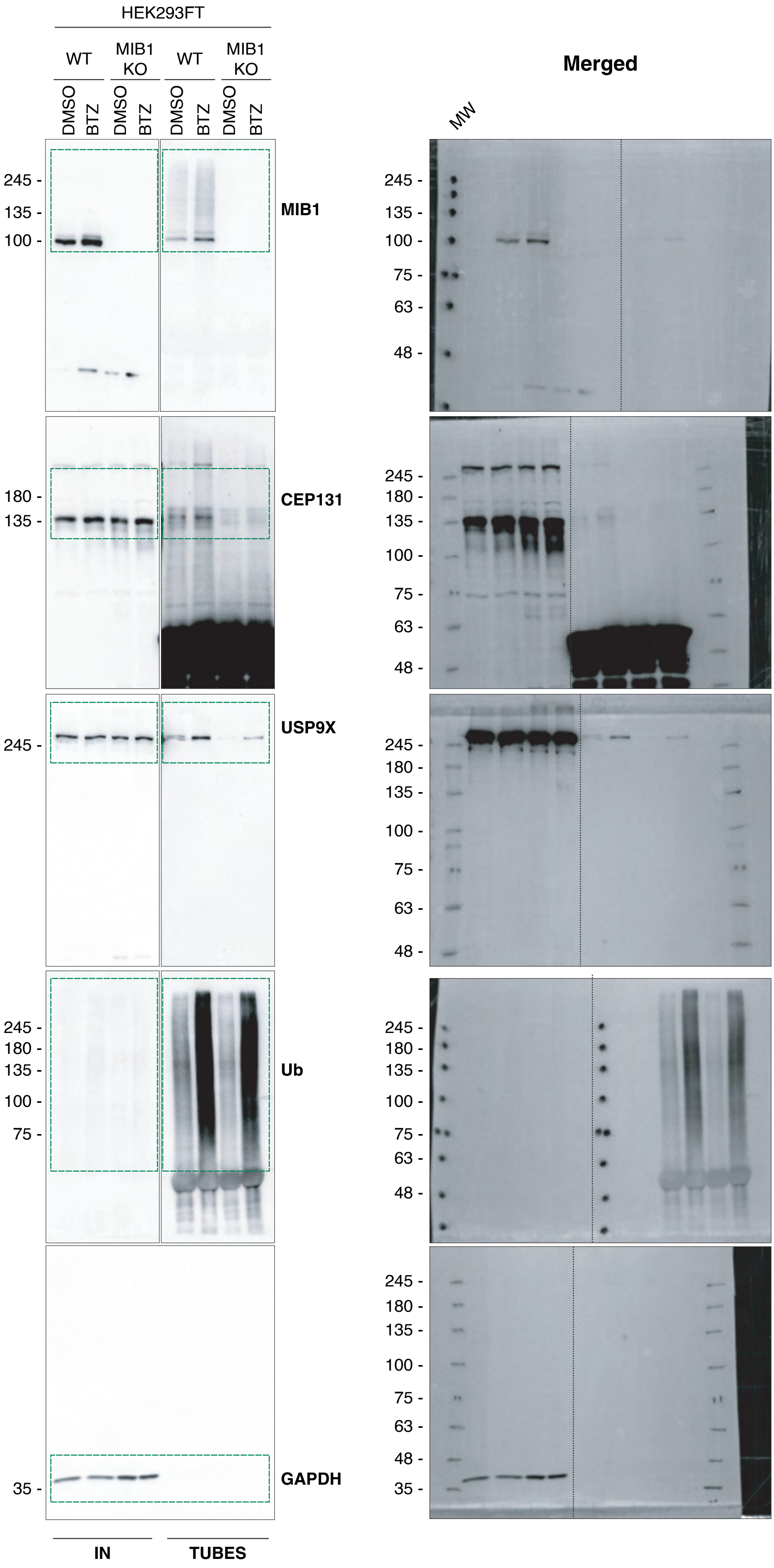

Supplementary Fig. 11c

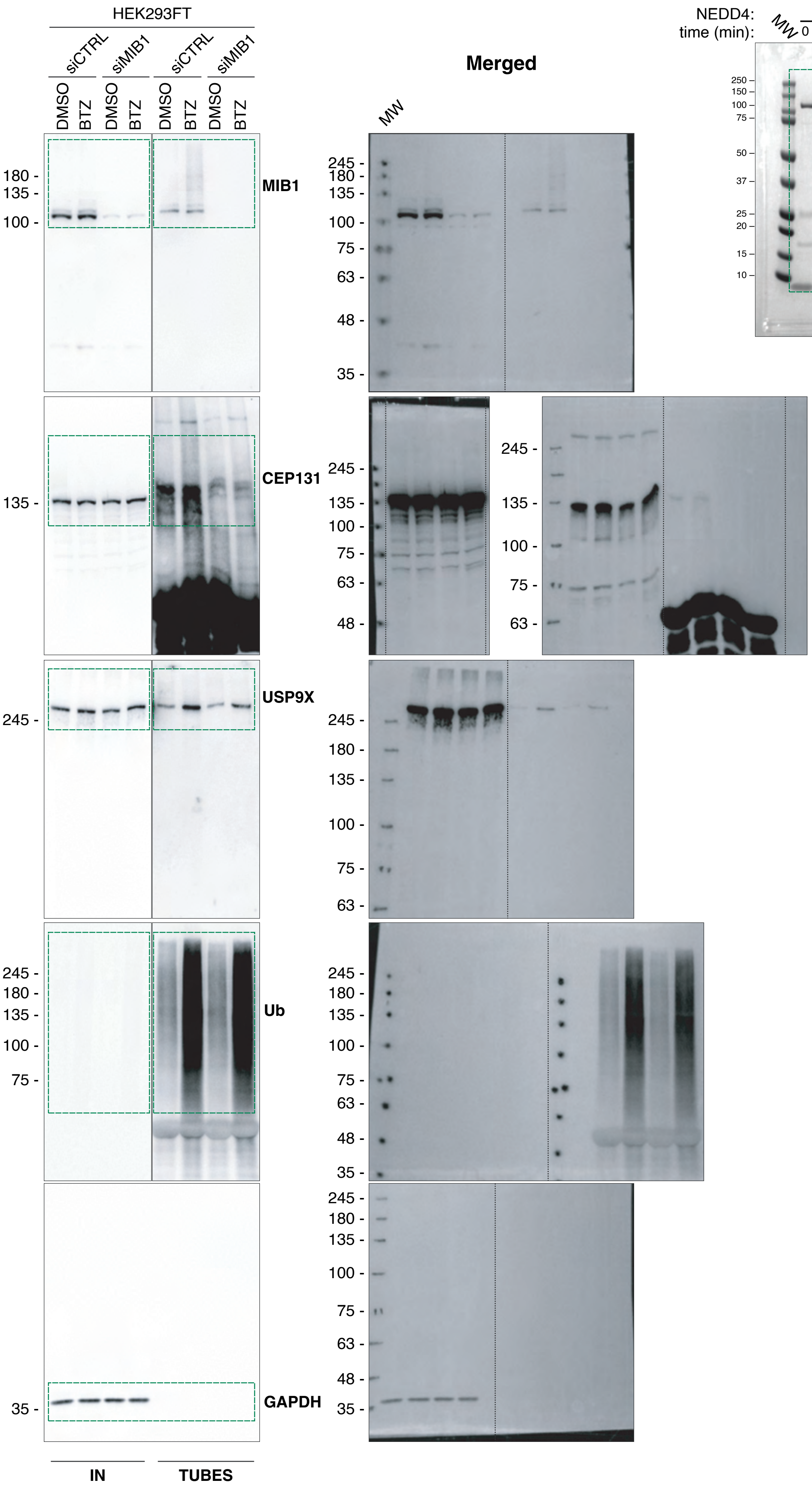

Supplementary Fig. 11d

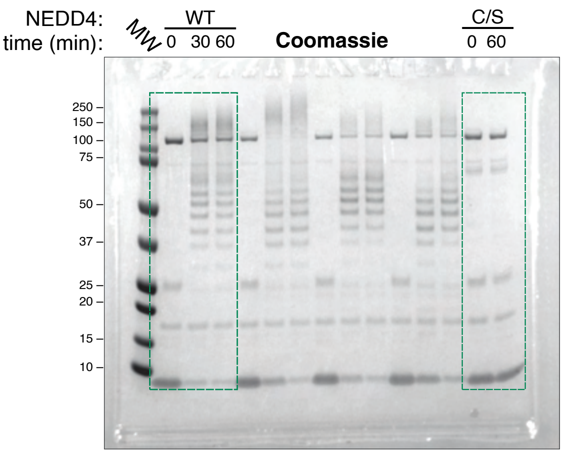

Supplementary Fig. 11e

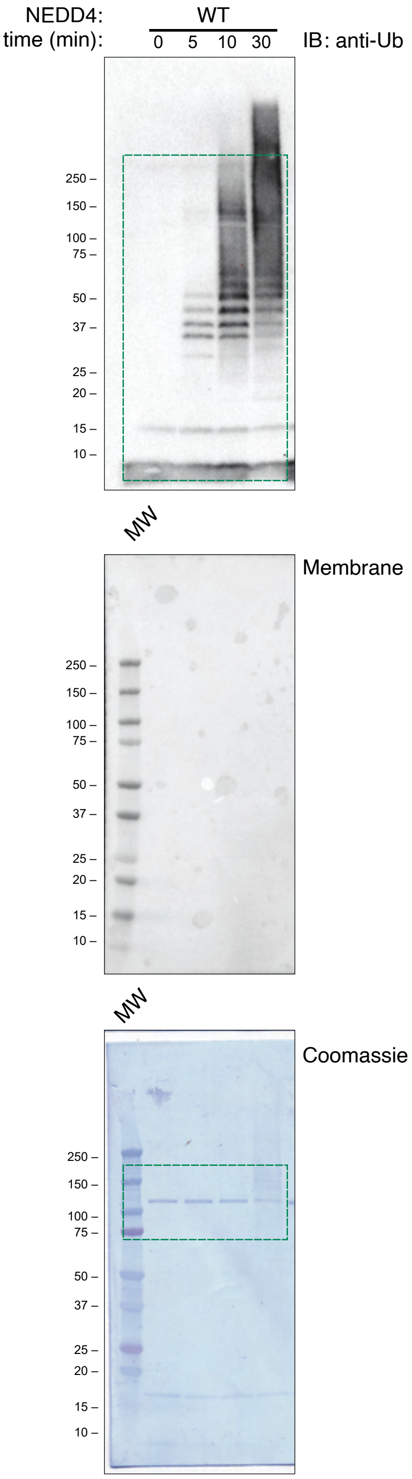

Supplement: Supplementary file 15 — Source Data [file 41467_2023_43326_MOESM15_ESM.zip › Source_Data_File/Uncropped_blots/Uncropped_blots.pdf]
