# Supplementary figures and images for: BioE3 identifies specific substrates of ubiquitin E3 ligases
Source: Nat Commun. 2023 Nov 23;14:7656. doi: 10.1038/s41467-023-43326-8 (PMC10667490; doi:10.1038/s41467-023-43326-8)

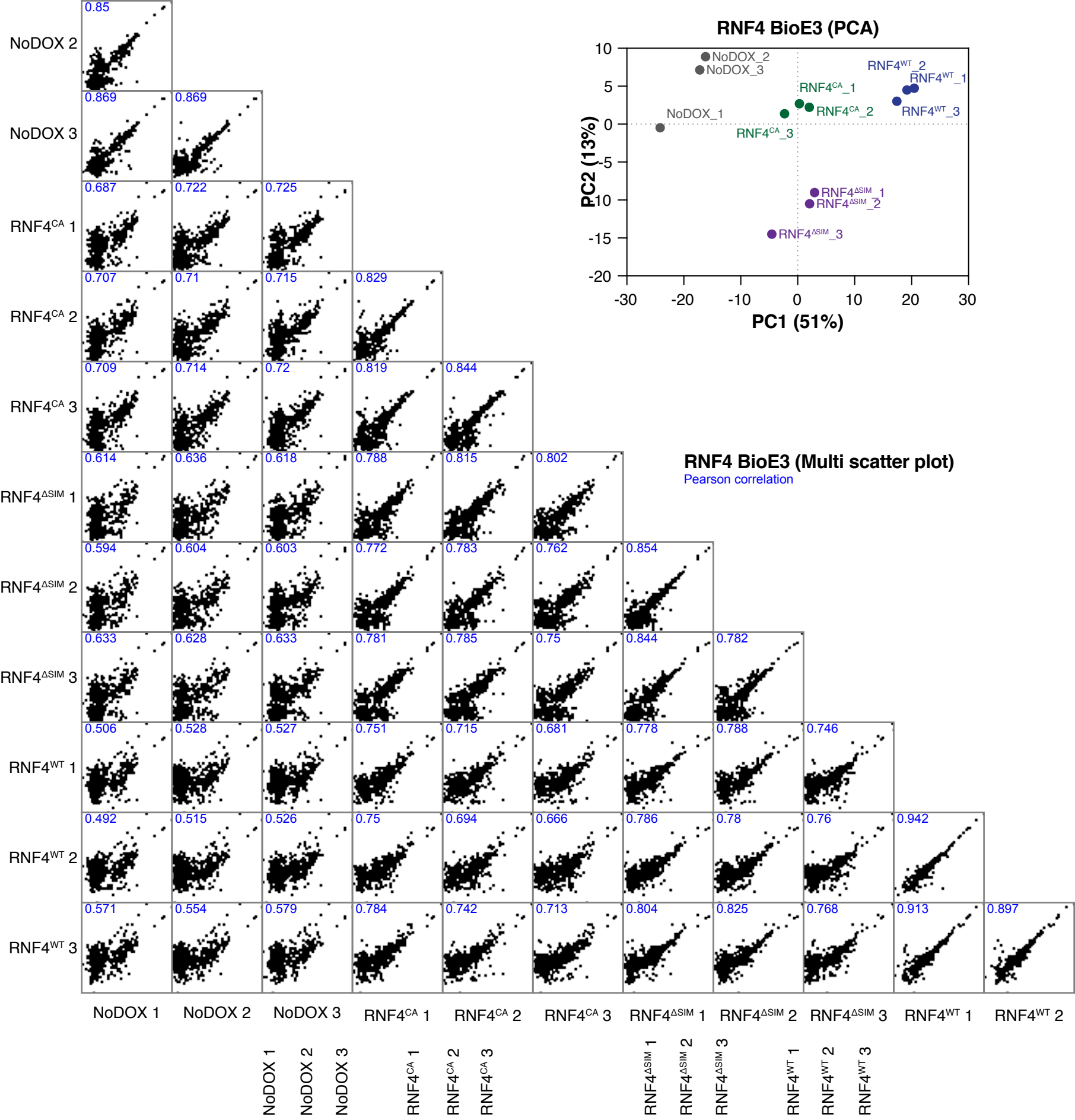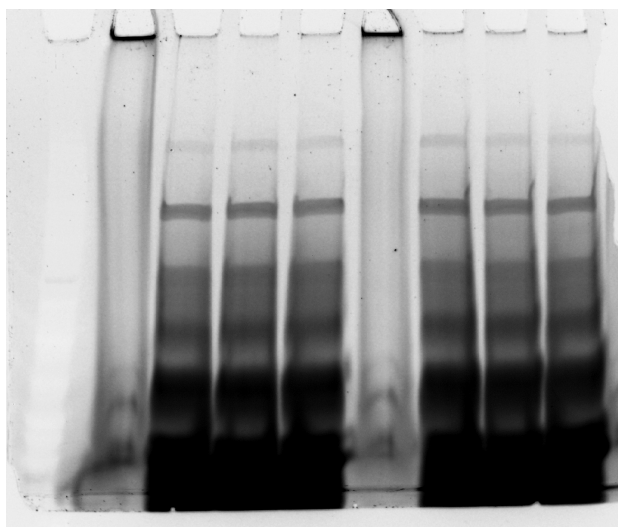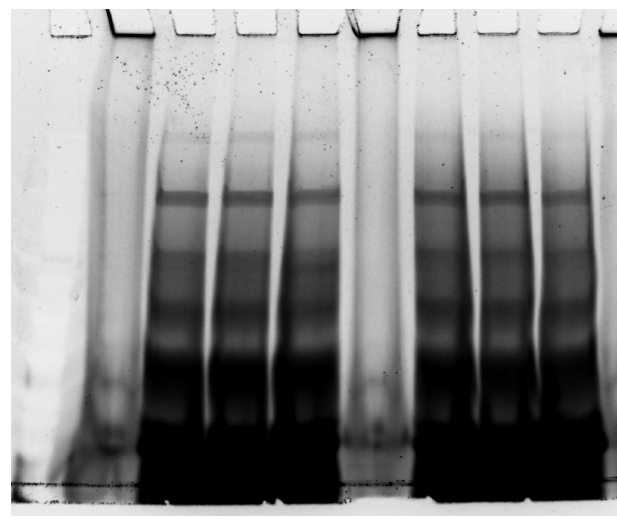

**SYPRO**

Supplement: Supplementary file 15 — Source Data [file 41467_2023_43326_MOESM15_ESM.zip › Source_Data_File/Fig_4a-b/Fig_4a-b_source_data.pdf]

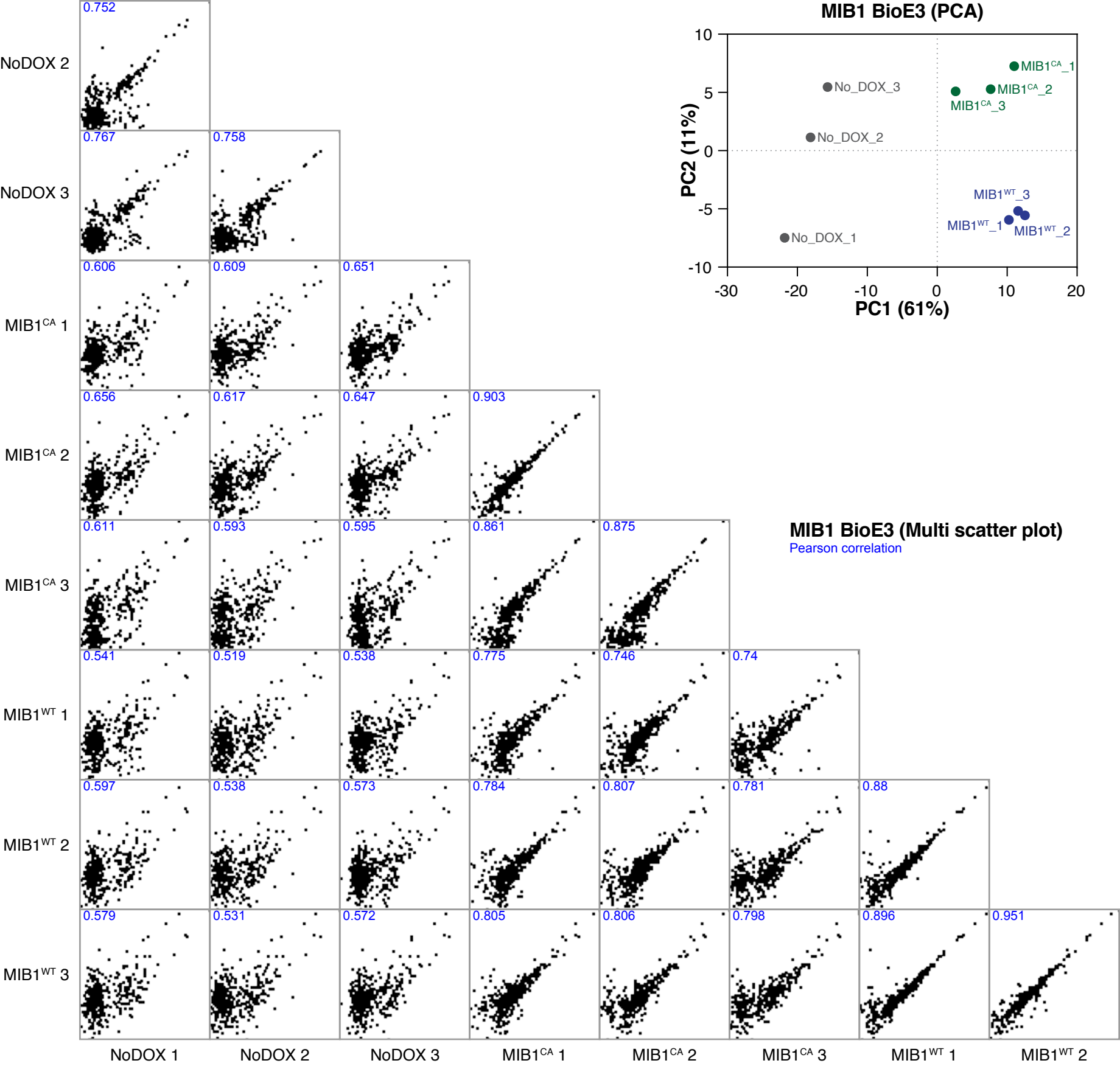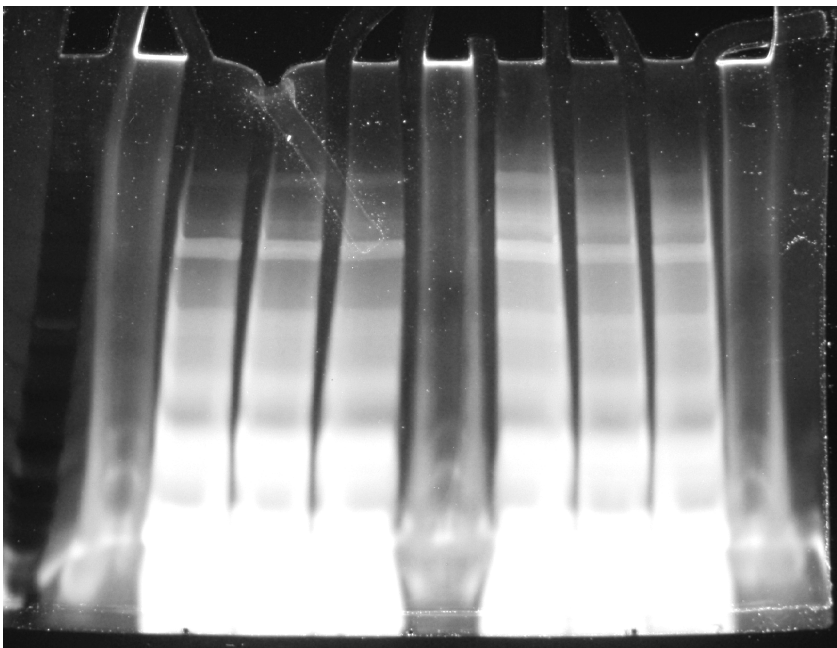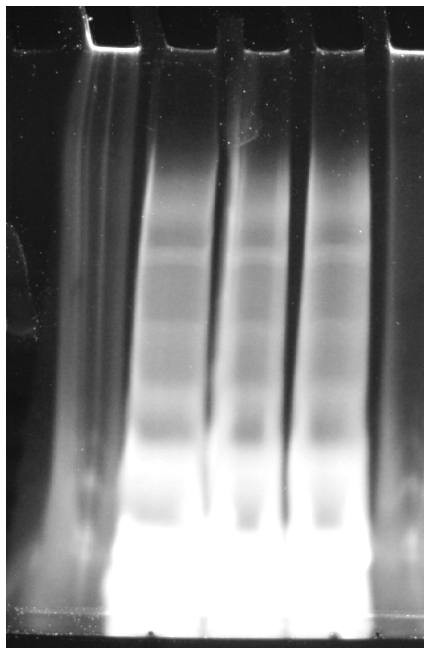

**SYPRO**

Supplement: Supplementary file 15 — Source Data [file 41467_2023_43326_MOESM15_ESM.zip › Source_Data_File/Fig_6c/Fig_6c_source_data.pdf]

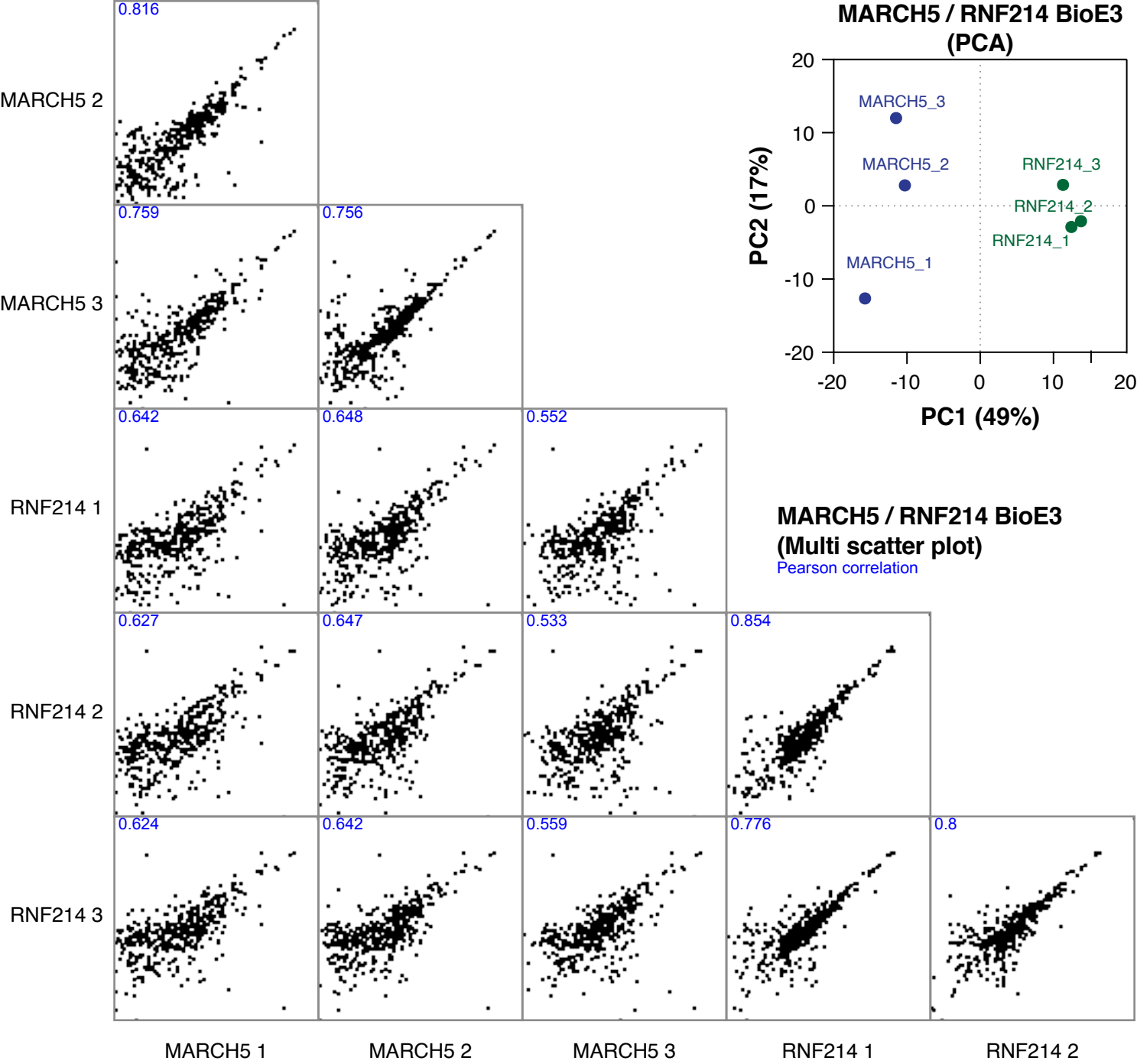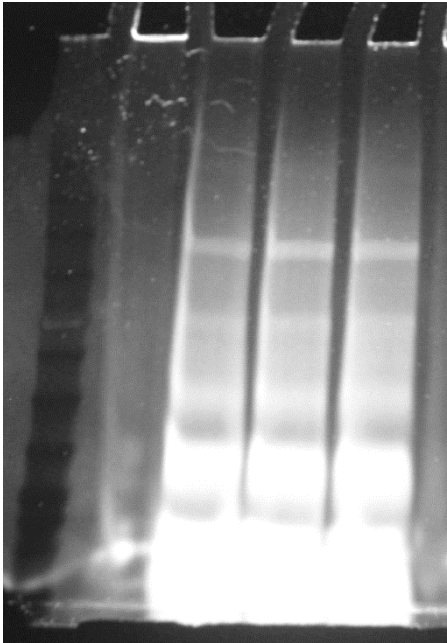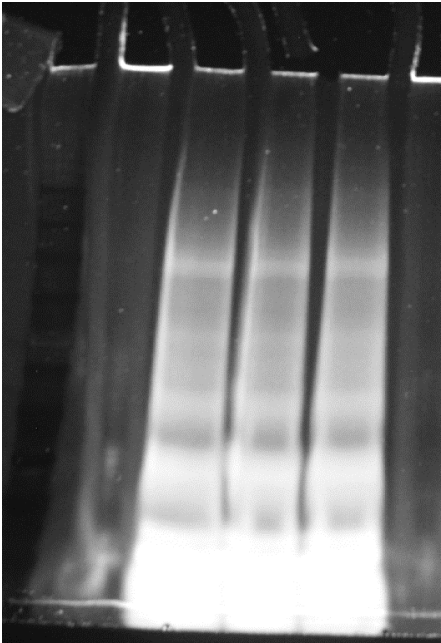

Supplement: Supplementary file 15 — Source Data [file 41467_2023_43326_MOESM15_ESM.zip › Source_Data_File/Fig_7e/Fig_7e_source_data.pdf]

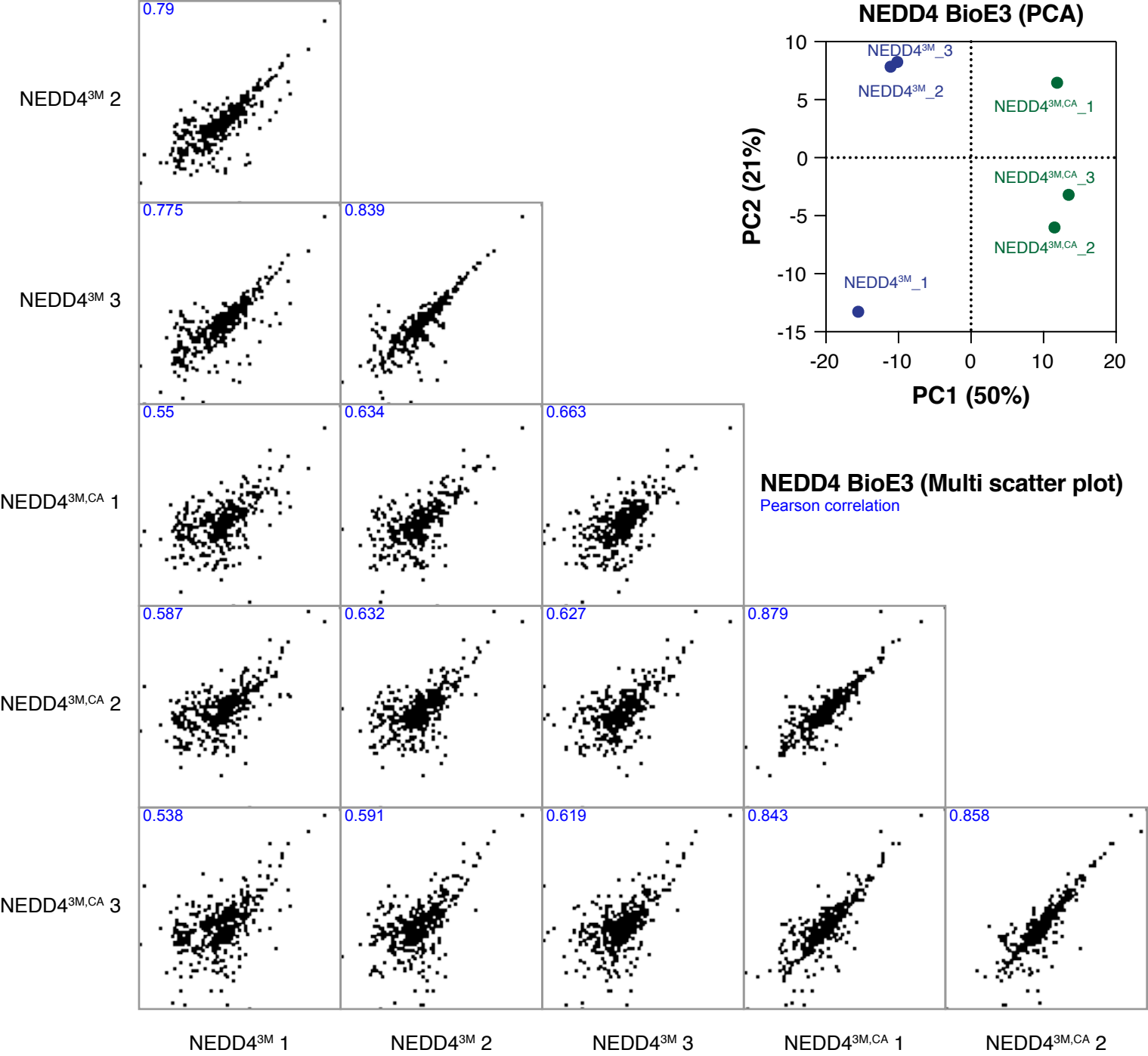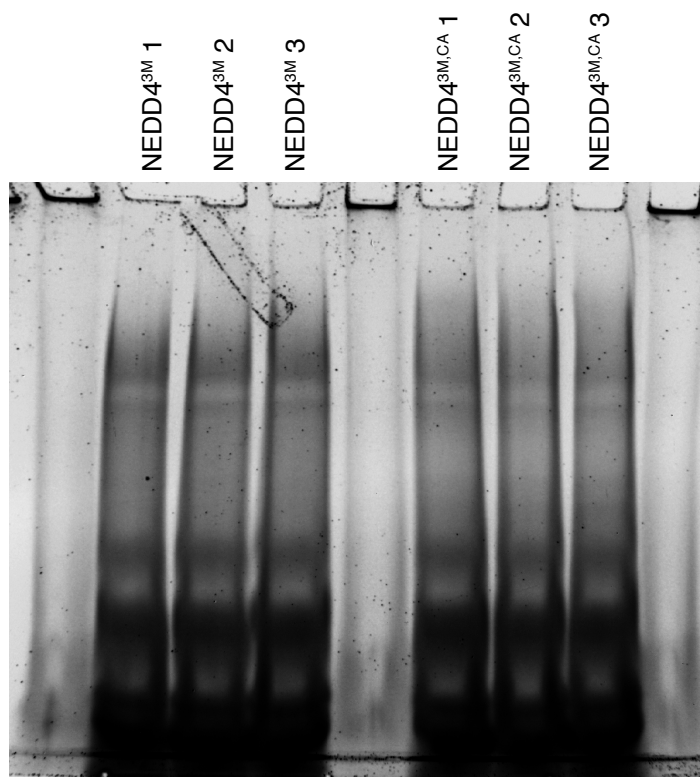

**SYPRO**

Supplement: Supplementary file 15 — Source Data [file 41467_2023_43326_MOESM15_ESM.zip › Source_Data_File/Fig_8g/Fig_8g_source_data.pdf]
